# Metabolomic heterogeneity of ageing with ethnic diversity: a step closer to healthy ageing

**DOI:** 10.1007/s11306-024-02199-8

**Published:** 2024-12-15

**Authors:** Dakshat Trivedi, Katherine A. Hollywood, Yun Xu, Fredrick C. W. Wu, Drupad K. Trivedi, Royston Goodacre

**Affiliations:** 1 Centre for Metabolomics Research (CMR), Department of Biochemistry, Cell, and Systems Biology, Institute of Systems Molecular and Integrative Biology, University of Liverpool, Liverpool, UK; 2 Clinical Metabolomics Unit (CMU), Human Development and Health, Institute of Developmental Sciences, University of Southampton, Southampton, UK; 3 Manchester Institute of Biotechnology (MIB), School of Chemistry, University of Manchester, Manchester, UK; 4 Andrology Research Unit (ARU), Division of Endocrinology, Diabetes and Gastroenterology, School of Medical Sciences, Faculty of Biology, Medicine and Health, University of Manchester, Central Manchester University Hospitals NHS Foundation Trust, Manchester, UK

**Keywords:** Ethnicity, Healthy ageing, Metabolomics, Mass spectrometry, Serum

## Abstract

**Introduction:**

Outside of case–control settings, ethnicity specific changes in the human metabolome are understudied especially in community dwelling, ageing men. Characterising serum for age and ethnicity specific features can enable tailored therapeutics research and improve our understanding of the interplay between age, ethnicity, and metabolism in global populations.

**Objective:**

A metabolomics approach was adopted to profile serum metabolomes in middle-aged and elderly men of different ethnicities from the Northwest of England, UK.

**Methods:**

Serum samples from 572 men of White European (WE), South Asian (SA), and African-Caribbean (AC) ethnicities, ranging between 40 and 86 years were analysed. A combination of liquid chromatography (LC) and gas chromatography (GC) coupled to high-resolution mass spectrometry (MS) was used to generate the metabolomic profiles. Partial Least Squares Discriminant Analysis (PLS-DA) based classification models were built and validated using resampling via bootstrap analysis and permutation testing. Features were putatively annotated using public Human Metabolome Database (HMDB) and Golm Metabolite Database (GMD). Variable Importance in Projection (VIP) scores were used to determine features of interest, after which pathway enrichment analysis was performed.

**Results:**

Using profiles from our analysis we classify subjects by their ethnicity with an average correct classification rate (CCR) of 90.53% (LC–MS data) and 85.58% (GC–MS data). Similar classification by age (< 60 vs. ≥ 60 years) returned CCRs of 90.20% (LC–MS) and 71.13% (GC–MS). VIP scores driven feature selection revealed important compounds from putatively annotated lipids (subclasses including fatty acids and carboxylic acids, glycerophospholipids, steroids), organic acids, amino acid derivatives as key contributors to the classifications. Pathway enrichment analysis using these features revealed statistically significant perturbations in energy metabolism (TCA cycle), *N*-Glycan and unsaturated fatty acid biosynthesis linked pathways amongst others.

**Conclusion:**

We report metabolic differences measured in serum that can be attributed to ethnicity and age in healthy population. These results strongly emphasise the need to consider confounding effects of inherent metabolic variations driven by ethnicity of participants in population-based metabolic profiling studies. Interpretation of energy metabolism, *N*-Glycan and fatty acid biosynthesis should be carefully decoupled from the underlying differences in ethnicity of participants.

**Supplementary Information:**

The online version contains supplementary material available at 10.1007/s11306-024-02199-8.

## Introduction

The global population is rapidly ageing, and man is expected to live longer than ever before. Countries globally will face increased old age dependency ratios (OADR) with the proportion of 60 years old set to double by 2050 (WHO, 2022). The question: ‘What is ageing?’—is yet to be fully answered (Kondoh et al., 2020). Despite substantial advances in understanding the mechanisms of aging, the translation of these insights from research to clinical application has been markedly constrained. This constraint reflects our limited understanding of the inherent variability in aging, especially in the context of the diverse environmental exposures, known as the exposome, that uniquely affect each individual. (Orešič et al., 2020). The heterogeneity impacts at all stages of life right from infancy to old age. For instance, rates of infant and maternal mortality (Knight et al., 2009), incidences of cardiovascular disease (CVD) (Lip et al., 2007), and the risk of type 2-diabetes (T2DM) (Mikhail et al., 2021) are all known to be higher in Black and South Asian populations globally. In comparison White counterparts suffer a higher mortality from cancer (Delon et al., 2022), dementia and Alzheimer incidences (Mukadam et al., 2023). From historical reports of selective immune response to fatal diseases such as Tuberculosis (TB), acquired immune deficiency syndrome (AIDS) (Winkler et al., 2004) and malaria (Arama et al., 2015) to more recent COVID-19 pandemic these disparities have been exemplified across ethnicities globally through evidently disproportionate impact and higher mortality rates (Tai et al., 2022; Yaya et al., 2020). Some studies refer to these as being a consequence of ‘community-level deprivation’ (Lo et al., 2021) whilst other propose ‘randomness’ to account for it (Smith, 2011). These disparities could be a result of genetics, environment, or an interaction between the two through epigenetic mechanisms. Independent of the cause, these disparities in health and disease must be investigated and understood to enable our populations to remain healthier, happier, and functional for longer. This becomes pivotal as we transition from the era of omics-based discoveries to precision medicine-led treatments and prognosis (Trivedi et al., 2017).

Ageing and ethnicity are two distinct entities with a common facet of being nonmodifiable and irreversible factors. Moreover, these are impacted by cultural, genetic, environmental, and socio-economic contributors. Although ethnicity can be considered a greater and more significant factor of ageing given its role within a population. Diverse ethnicities can exhibit varying preferences and awareness towards diet and nutrition which can reflect in their metabolome (Clarke et al., 2023) and can be associated to diet related metabolic diseases (Stratakis et al., 2022). Additionally, contributions from non-nutrient components like their choice of medical approach, i.e., choosing traditional, herbal, homeopathic, or naturopathic practices over modern medicine, this too can determine their metabolic makeup. There is evidence for geographical location and migration to play a role in cluster genetic variations within a population (Tishkoff & Kidd, 2004). Thus, understanding ageing through ethnic phenotype could be a way forward. Despite ageing being independent of gender distinctions, our knowledge of hormonal and biochemical processes at the onset of ageing in men is relatively understudied (Gava et al., 2011; Lee et al., 2009) in contrast to that in females (Huddleston et al., 2011). Whilst both ethnicity and age have been studied as factors in such studies, their influence on changes in metabolome in an ageing, multi-ethnic population remains unexplored.

In recent years, metabolomics has emerged as a powerful investigation tool in simplifying complex interplay between genetics, environment, and health. These phenotyping approaches have been used to study mammalian systems and changes occurring at the cellular level due to diseases, drug dosage and other factors like nutrition or environmental perturbations. Metabolomics which does not target predefined sets or lists of metabolites has immense potential when probing a system to generate a hypothesis besides its clinical utility in diagnosis, phenotypic stratification and as a precursor to the development of targeted metabolic therapies. Mass spectrometry-based methods have been tested for phenotyping large populations (Dunn et al., 2011, 2015; Hajjar et al., 2023; Psychogios et al., 2011) given the high-resolution and high throughput capabilities. The findings of these studies form a baseline reference of serum metabolite levels in a relatively healthy population and, to some extent, explore the complex relationships among demographic factors, environmental influences, and disease-associated heterogeneity and metabolomics.

Despite the perceived sample preparation and chromatographic challenges, LC–MS has been the versatile choice over the years for high-resolution data acquisition (Roberts et al., 2022; Trivedi, 2012; Vuckovic 2012). The addition of GC–MS extends the detection coverage to small polar molecules which are useful as these represent central carbon and nitrogen metabolism ( McGarrah et al., 2018; Misra, 2021; Mojsak et al., 2021; Zeki et al., 2020). In this study we use a combination of reversed phase LC–MS and GC–MS platforms to generate metabolomic profiles from human serum for comprehensive coverage (Zeki et al., 2020).

## Materials and methods

### Study population

The study population consisted of middle-aged and older men (mean ± SD age: 59 ± 12 years) of White European, South Asian, and African Caribbean ethnic origins. These men were originally recruited from the Manchester area for the Human Serum Metabolome project (Dunn et al., 2015) and the European Male Ageing Study (EMAS). Briefly, fasting blood samples were collected along with clinical parameters (including age, gender, BMI, smoking status), health questionnaire and anthropometric measures. Details including sample collection and background have been outlined by Lee et al. (2009) and Dunn et al. (2015). From 709 samples available those with missing metadata such as age, Body Mass Index (BMI), comorbidities or with ‘missing’ or ‘other’ ethnicities were excluded (see details in Table S1). A subset of 572/709 samples analysed in this study are detailed in Table 1. Individuals were matched for age and BMI. Individuals with more than three co-morbidities reported were excluded to avoid confounded effects due to multimorbidity (Salisbury et al., 2011) in the profiles generated.

**Table 1:** Distribution and demographics of subjects in this study

Parameters	White European (WE)	South Asian (SA)	African Caribbean (AC)
*N*	287	143	142
Age (years)	63.66 ± 10.68	56.97 ± 11.12	54.01 ± 10.63
BMI (kg/m^2^)*	27.47 ± 3.89	27.75 ± 3.70	27.80 ± 4.86
Alcohol intake	74: 141 (0.34)	141:2 (70.5)	128:14 (9.14)
Smoking status	21: 266 (0.07)	22: 120 (0.18)	21: 121 (0.17)
Morbid: healthy**	150: 137 (1.09)	86: 57 (1.5)	60: 82 (0.73)

*BMI and age are expressed as mean ± standard deviation**.** Alcohol intake is expressed as a ratio of drinkers: non-drinkers (72 of the 287 had missing data for alcohol consumption). Smoking status is expressed as a ratio of smokers: non-smokers. **Data were collected on morbidities including high blood pressure, heart conditions, liver conditions, kidney conditions, thyroid disease, prostate disease, diabetes, bronchitis, asthma and incidences of any cancers and stroke. Subjects with 2 or more comorbidities of these were classed as morbid, whilst those with 0 or 1 of these conditions reported were classed as healthy

### Chemicals and reagents

For *LC–MS:* Optima^®^ LC–MS grade water, methanol, and formic acid (Fisher Scientific) and for *GC–MS:* Optima^®^ LC–MS grade water, anhydrous pyridine, *N*-methyl-*N*-trimethylsilyl tri fluoroacetamide (MSTFA), and methoxamine (Fisher Scientific). For retention index (RI) reagents used were decane, dodecane, pentadecane, nonadecane, docosane and hexane. Internal standard (IS) mix consisted of lysine-*d*_4_, succinic-*d*_4_ acid, glycine-*d*_5_, and benzoic acid-*d*_5_ (Sigma Aldrich). HPLC grade absolute ethanol (99.8%, *v/v*) diluted in water was used for apparatus cleaning and general maintenance.

### Sample preparation

Samples were stored at − 80 °C until preparation. Samples were randomly picked and further re-randomised prior to data acquisition to ensure no systematic biases remained. Samples were passively thawed on ice during preparation. Two × 50 µL aliquots per sample, one for each LC–MS and GC–MS analysis were collected into a 1.5 mL Eppendorf tubes. For GC–MS analysis 50 µL of IS was added to all samples at this stage. Serum was deproteinised by adding 200 µL ice-cold methanol. Samples for LC–MS analyses were vortexed for 10 s followed by centrifugation at 30 °C, 13,500 *g,* for 15 min. The supernatant was lyophilised in a vacuum centrifuge overnight. Lyophilised pellets were reconstituted in 100 µL methanol. Samples were vortexed, transferred to glass vials for LC–MS analysis.

GC–MS derivatisation: Post-deproteinisation samples were derivatised with addition of 25 µL *O*-methoxyamine-HCl in anhydrous pyridine followed by vortex mixing for 10 s and placing onto a heat block at 65 °C for 40 min. Following that, 25 µL of MSTFA was added to each sample, vortex mixed (10 s) and placed on to a heating block at 65 °C for 40 min. 10 µL of 1 mg/mL RI solution (Dunn et al., 2011) was added to each vial before vortex mixing for 10 s and centrifuging at 30 °C, 17,500 *g* for 15 min. 50 µL of supernatant was transferred to a sterile GC glass vial capped with rubber sealed caps.

Quality control: A fixed amount of serum was aliquoted from all samples to prepare a pooled quality control (QC) and system conditioning QCs as described in (Broadhurst et al., 2018). Extraction blanks were prepared alongside samples by substituting serum volume (50 µL) with water. Solvent blanks were also prepared to assess data quality and system performance but not used in statistical analysis. Pooled QCs were used during acquisition and pre-processing steps to assess instrument and data quality.

### Metabolic profiling and data acquisition

LC–MS analysis: Samples were analysed with an Ultimate 3000 UHPLC (Thermo Scientific) coupled to a Q-Exactive™ Plus Hybrid Quadrupole-Orbitrap™ Mass Spectrometer. Hypersil GOLD C_18_ column (1.9 µm, 2.1 mm × 100 mm), heated and held at 55 °C was setup for chromatographic separation of analytes. 5 µL extracted serum was injected for each sample. Metabolites were separated in samples at a flow rate of 0.5 mL/min with a controlled 15-min gradient of LC–MS grade mobile phases: (A) H_2_O with added (0.1% (*v/v*) formic acid) and (B) methanol (MeOH) with (0.1% (*v/v*) formic acid). The gradient started at 5% (B) for 2 min, increased linearly to 95% (B) over 8 min, and was held for 2 min. It then returned to 5% (B) at 12.1 min, held for 2 min and finally re-equilibrated at 14.1 min to initial conditions.

Data acquisition was conducted in full MS mode in the scan range of 70–1050 m*/z* with a resolution of 70,000 FWHM, an AGC target of 3.10^6^ and a maximum injection time of 200 ms. The samples were analysed in positive mode. For MS, the spray voltage was set to 3.5 kV. The capillary temperature was 260 °C, sheath gas 50, aux gas 12.5, max spray current 100 and the probe heater temperature was 425 °C for the analysis. Tuning, calibration, and instrument maintenance was carried out as per the manufacturer’s recommendations.

GC–MS analysis: Data were acquired using an Agilent 7890B GC paired with an Agilent 5977B MSD and operated with an Agilent 7693 autosampler. The sample (1 μL) was injected onto a VF-5 ms (inert, 5% phenylmethyl polysiloxane) column (30 m × 250 μm × 0.25 μm; Agilent Technologies) with an inlet temperature of 280 °C and a split ratio of 25:1. Helium was used as the carrier gas with a flow rate of 1.5 mL/min and a pressure of 14.1 psi. The chromatography was programmed to begin at 70 °C with a hold time of 4 min, followed by an increase to 300 °C at a rate of 14 °C/min and a final hold time of 4 min. The total run time per analysis was 14.42 min. The MS was equipped with an electron ion source using 70 eV ionisation and a fixed emission of 35 μA. The mass spectrum was collected for the range of 50–550 *m/z*.

### Data processing and statistical analysis

Data pre-processing: All raw data files were centroided and converted to.*mzXML* using ProteoWizard 3.0 (Chambers et al., 2012). Data were deconvolved and aligned using Progenesis QI for LC, and *eRAH* package using R for GC combined with in-house data pre-processing toolbox [available at BioSpec Github (https://github.com/Biospec/)]. For LC–MS, parameters were setup within data pre-processing method for all 30,265 features detected. A filter was applied such that if a feature was not present in at least 10% of QCs, it was removed, and remainder of features were kept only if relative standard deviation (%) was less than coefficient of variance (%CV) of 40%. This was followed by blank signal intensity ratio filter, which removed 28,507 features. The resulting output had 1758 features. Similar strategy for GC-MS reduced 2074 features to 1253. Pooled QCs were used to algin signal drifts within batches and to perform LOESS (locally estimated scatterplot smoothing) normalisation between batches. The data were log_10_-transformed, Pareto scaled, and missing values replaced with cubic spline interpolation.

Clustering analysis: Data trends were tested with the unsupervised multivariate method of Principal Component Analysis (PCA) using MetaboAnalyst 4.0 (Chong et al., 2019).

Discriminant analysis and classification modelling*:* To test the classification and prediction ability of the LC–MS and GC–MS profiles generated we employed supervised approach with PLS-DA based classification models for: (i) age with the four intervals 40–49.99, 50–59.00, 60–69.99 and ≥ 70 years of age; (ii) age < 60 and ≥ 60 years; and (iii) the three ethnicities (South Asian, White European and African Caribbean). These models were validated with bootstrap resampling with 1000 iterations. In addition, 1000 null models were also generated where the output class variable was randomly permuted (Gromski et al., 2015). PLS-DA was performed in MATLAB 2019a (MathWorks), and the results are presented as correct classification rates (CCRs) for the 1000 test sets only.

Feature selection and ranking: To identify the significant contributors to the PLS-DA classifications above the variable importance in projection (VIP) scores were calculated using MATLAB 2019a for all three models. The top features from LC–MS analysis were putatively annotated using accurate mass matching to HMDB (https://hmdb.ca). For GC–MS, the features were assigned putative identifications by matching fragment spectra to compound spectra in the GMD (http://gmd.mpimp-golm.mpg.de/). Metabolomics Standards Initiative (MSI) guidelines were adhered to for annotation of all compounds (Sumner et al., 2007) and so LC–MS were at Level 3 at best and GC–MS to MSI Level 2.

Pathway enrichment and functional analysis: Mummichog was employed within MetaboAnalyst 4.0 (https://www.metaboanalyst.ca) (Pang et al., 2022) to assess collective functional impact of significant metabolic features (Li et al., 2013). For mummichog analysis all *m/z *features were ranked by Mann-Whitney (MW) U-test and Kruskal Wallis (KW) ANOVA scores, and corresponding *p*-values. These features were then mapped onto a combination of known human metabolic models, including Kyoto Encyclopedia of Genes and Genomes (KEGG) (Kanehisa & Goto, 2000), Biochemical Genetic and Genomic knowledgebase (BiGG) (King et al., 2016) and the Edinburgh Model (Ma et al., 2007).

Correlation and confounder analysis: To identify associations and guide downstream statistical analysis, a Spearman’s correlation test was performed. We aimed to investigate association of age and ethnicity to the metadata available for these subjects. The metadata included anthropometric measures (height, BMI, waist circumference, waist-to-hip and waist to height ratios), clinical measurements (glucose, insulin, homa_ir (homeostatic model assessment of insulin resistance), homa_s (homeostatic model assessment for insulin sensitivity), shbg (sex hormone binding globulin), cholesterol, LDL (low density lipoprotein) and HDL [high density lipoprotein) cholesterol, triglycerides, HDL (high density lipoprotein) triglycerides, high_bp (blood pressure)] and data on comorbidities and health conditions (bronchitis, asthma, liver condition, kidney condition, thyroid, heart condition, diabetes, prostate condition, cancer and stroke). A correlation matrix was calculated for these in R and visualised as a correlation squares plot (Fig. S8). To evaluate confounding effects of age in ethnicity classification and vice versa we built PLS-DA models that were age-adjusted in ethnicity or ethnicity-adjusted in age. Additionally, models were created using subsets of our subject population i.e., drinkers versus non-drinkers to test for influence of alcohol intake in our classification models. Using the same subsets pathway enrichment analysis was also performed to determine confounding effect of alcohol intake in age specific pathway impact. To determine if there was a confounding effect of ethnicity on citric acid expression in our cohort, box whiskers plots and pairwise *t-*test was performed on normalised citrate intensities of these subjects.

## Results

The metabolic features which passed data filtering thresholds, QC normalisation, alignment checks and appeared consistently for all samples were deemed reproducible. We retained 1758 such features from LC–MS and 1253 such features from GC–MS for statistical analysis. PCA showed no clear clustering or trends in the data separating these samples by age or ethnicity, however, QCs showed good clustering (Fig. S1). No confounding effects were seen on the age and ethnicity models constructed. No significant correlation was observed between age, ethnicity, and subject metadata from Spearman’s correlation tests. Figure S9 displays insignificant difference in citrate level across ethnicities.

### Classification of subjects by *ethnicity*

**Fig. 1:**
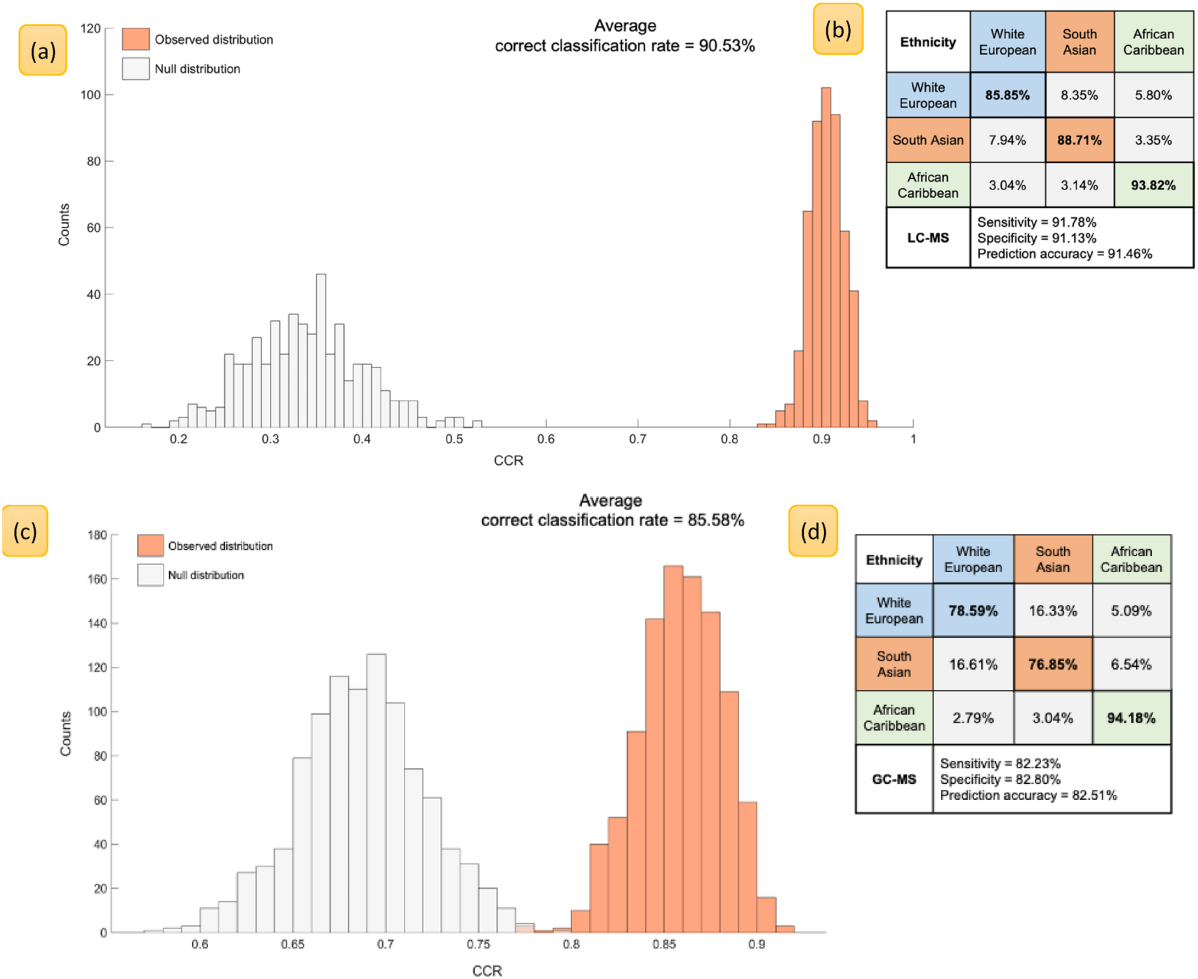
PLS-DA models built to classify subjects based on their ethnicity (White European vs. South Asian vs. African Caribbean) using metabolic features discovered with LC–MS (**a**, **b**) and GC–MS (**c**, **d**). Histograms **a** and **c** show the null distribution (grey bars) versus observed distribution (orange bars) the averaged correct classification and graphics **b** and **d** report class-wise classification rates, in addition to model’s overall sensitivity, specificity and prediction accuracy for each bootstrapped PLS-DA model

**Fig. 2:**
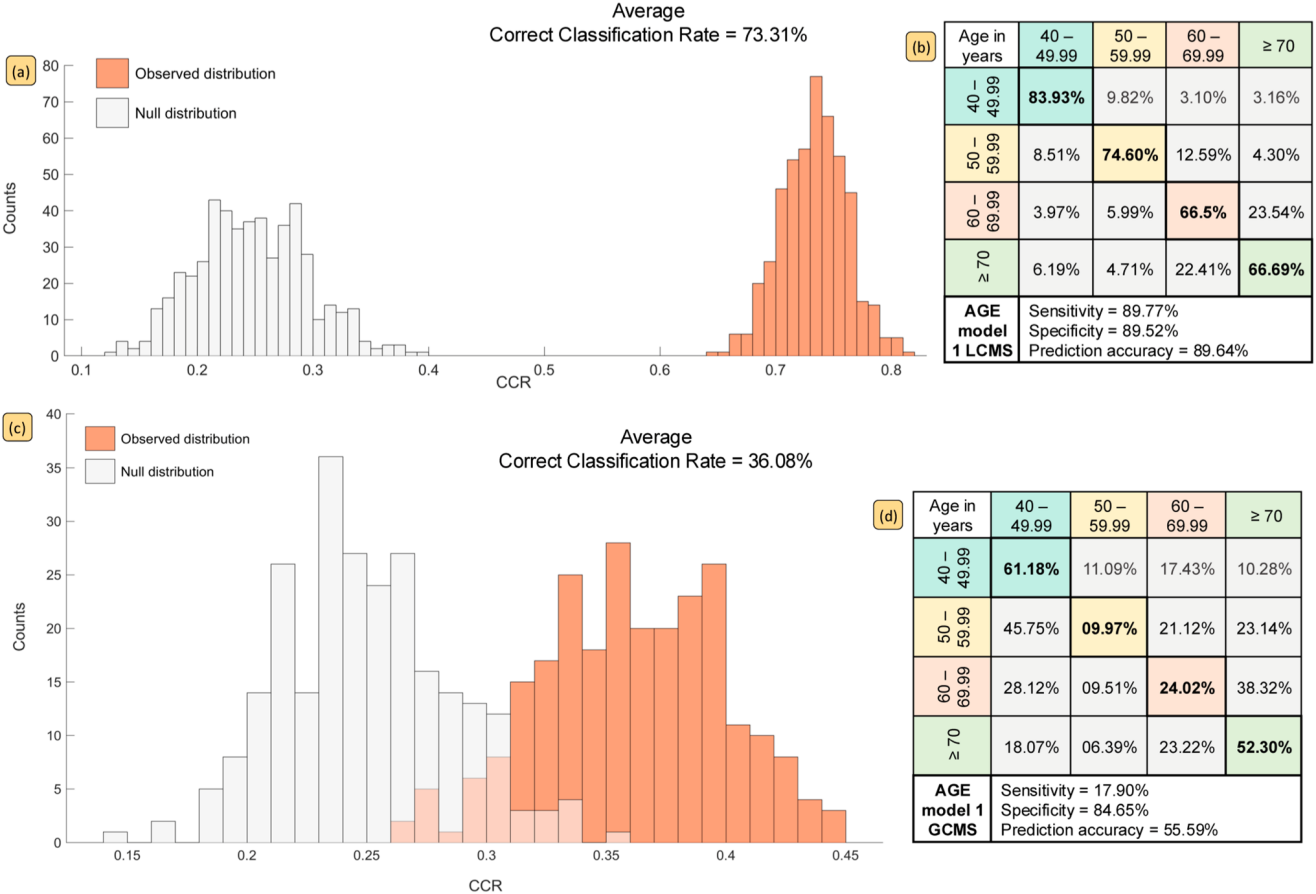
PLS-DA models built to classify subjects based on age in four brackets (viz. 40–49.99 years, 50–59.99 years, 60–69.99 years, or  ≥ 70 years) using metabolic features discovered with LC–MS (**a**, **b**) and GC–MS (**c**, **d**). Histograms **a** and **c** show the null distribution (grey bars) versus observed distribution (orange bars) the averaged correct classification and graphics **b** and **d** report class-wise classification rates, in addition to overall sensitivity, specificity and prediction accuracy for each bootstrapped PLS-DA model

**Fig. 3:**
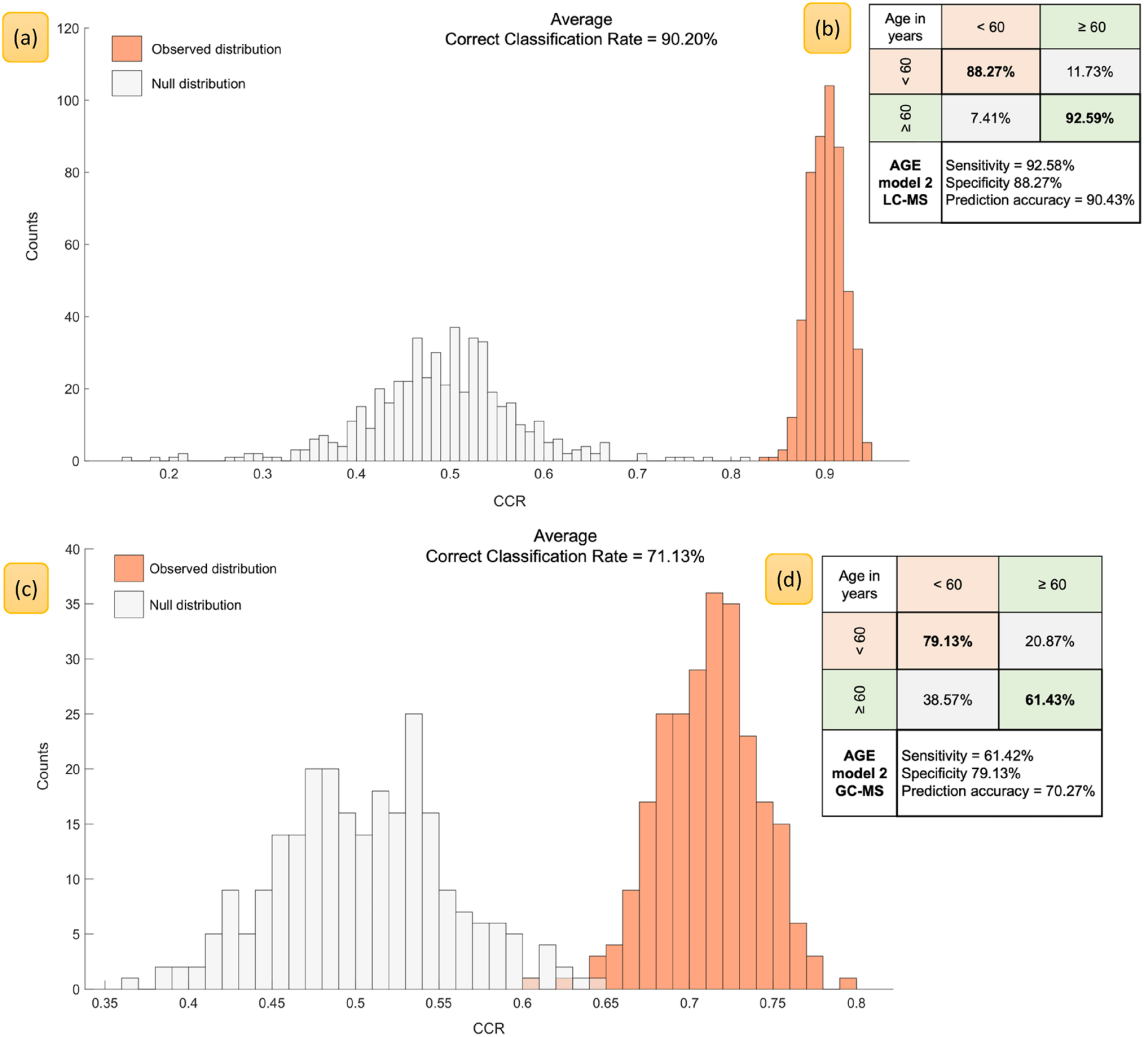
PLS-DA models built to classify subjects based on two classes of age (viz. < 60 years vs. ≥ 60 years old) using metabolic features discovered with LC–MS (**a**, **b**) and GC–MS (**c**, **d**). Histograms **a** and **c** show the null distribution (grey bars) versus observed distribution (orange bars) the averaged correct classification and graphics **b** and **d** report class-wise classification rates, in addition to overall sensitivity, specificity and prediction accuracy for each bootstrapped PLS-DA model

Samples were divided into three categorical classes based on subject’s ethnic origin. Classes consisted of *n* = 143 SAs, *n* = 142 ACs and *n* = 287 WEs. It is known that models generated using unbalanced sample numbers may introduce bias towards a single class i.e., WEs ethnicity outnumbering SAs and ACs approximately 2–1. To avoid this we applied the Synthetic Minority Oversampling Technique (SMOTE) previously reported (Chawla et al., 2002) to our dataset. We built and compared PLS-DA models built with and without SMOTE applied data. Models with SMOTE applied showed marginal but not statistically significant improvement in their averaged correct classification rate (data not shown), indicating that SMOTE did not bias these analyses to the smaller group. A series of PLS-DA models were built and rather than show PLS scores, which in the literature are at best generated from only the training data and do not represent the outputs of the models [in terms of the *Y*-variable that is used as the target output (Westerhuis et al., 2008)], we show our results in terms of the ability for PLS-DA to classify the test set data. Therefore, Fig. 1 represents a histogram of the test set outputs from 1000 bootstrap validated PLS-DA models built to classify subjects by their ethnicity using metabolic features in their serum. The figure shows observed versus null distributions (from 1000 permutation tests) and reports CCRs alongside prediction accuracy, sensitivity, and specificity of these models (the same process was used for Figs. 2 and 3).

### Classification of subjects by *age*

For classification by age, two models were built, each using different class distributions. In model 1, subjects were stratified into four 10-year bands between 40 and 86 years i.e., 40–49.99 years (*n* = 145), 50–59.99 years (*n* = 174), 60–69.99 years (*n* = 121) or ≥ 70 years (*n* = 140). In model 2, the subjects were split into two broader classes: < 60 years (*n* = 319) versus ≥ 60 years (*n* = 261). Figure 2 shows PLS-DA output and permutation testing distributions for model 1. Classifying subjects into tighter 10-year age bands, resulted in decreased CCRs of 73.13% (LC–MS) and 36.08% (GC–MS), indicating lesser metabolic variation in serum profiles. Alternatively, this may be due to the reduced number of samples within four groups compared to two group comparison. Both classification rates and prediction accuracies were seen to improve significantly for model 2 when just two age brackets are used, with almost no overlap between the actual models and the null distributions (Fig. 3).

### Determining confounding effects of age and ethnicity

The models created to test confounding effects of age in ethnicity and vice versa reported average correct classification rates of < 30% (for both instances). This indicated no obvious confounding effects were present Fig. S2. Additionally, PLS-DA classification for ethnicity showed no significant contribution from ‘age’ when included as a variable.

### Feature annotation and metabolite identification

Having established a clear and good classification of these men based on their metabolomic profiles, by age and ethnicity, the next stage was to attempt to understand what biochemical processes may be driving these models. We note here that we are aware of the published MSI guidelines (Sumner et al., 2007), where two orthogonal properties are used for annotation and identification (if a standard is available). For our analyses the two different physicochemical properties are retention time—relating to polarity in LC–MS and volatility in GC–MS—and mass spectrometry information, where for LC–MS this is solely accurate mass (due to the high mass resolution mass analyser used) and for GC–MS fragmentation patterns due to high energy (viz. 70 eV) electron ionization. Thus, for GC–MS our identification against external libraries (e.g., GMD) is at best MSI Level 2, and for LC–MS as we are using accurate mass only, this is less confident. In addition, we did fact check our annotations to make sure they are not spurious following suggestions published recently (Theodoridis et al., 2023). If an identification of analyte had low biological plausibility (i.e. a plant specific metabolite seen in serum) or low plausibility in terms of chromatographic retention (i.e. a lipid peak was seen within first two minutes of a gradient analysis using C_18_ column), we annotated the feature as ‘unknown/unidentified’.

To select discriminatory variables, we used input variables from PLS-DA models with VIP score > 1 from both ethnicity and the two age PLS-DA models. Features were ranked by descending VIP scores. The top 10% variables from each model were annotated with *m/z* matching to HMDB (LC–MS) and accurate mass and spectral fragmentation match to GMD database (GC–MS). In PLS-DA modelling a clear ranking-based feature selection may not always be possible when too many variables contribute equally towards classification of the response variable, therefore data specific cut-off should be calculated for accurate ranking of features (Akarachantachote et al., 2014). We chose the top 10% annotated variables (Tables S2–S5) ranked by their VIP scores for visualisation (Figs. S3–S7).

### Pathway enrichment analysis

**Fig. 4:**
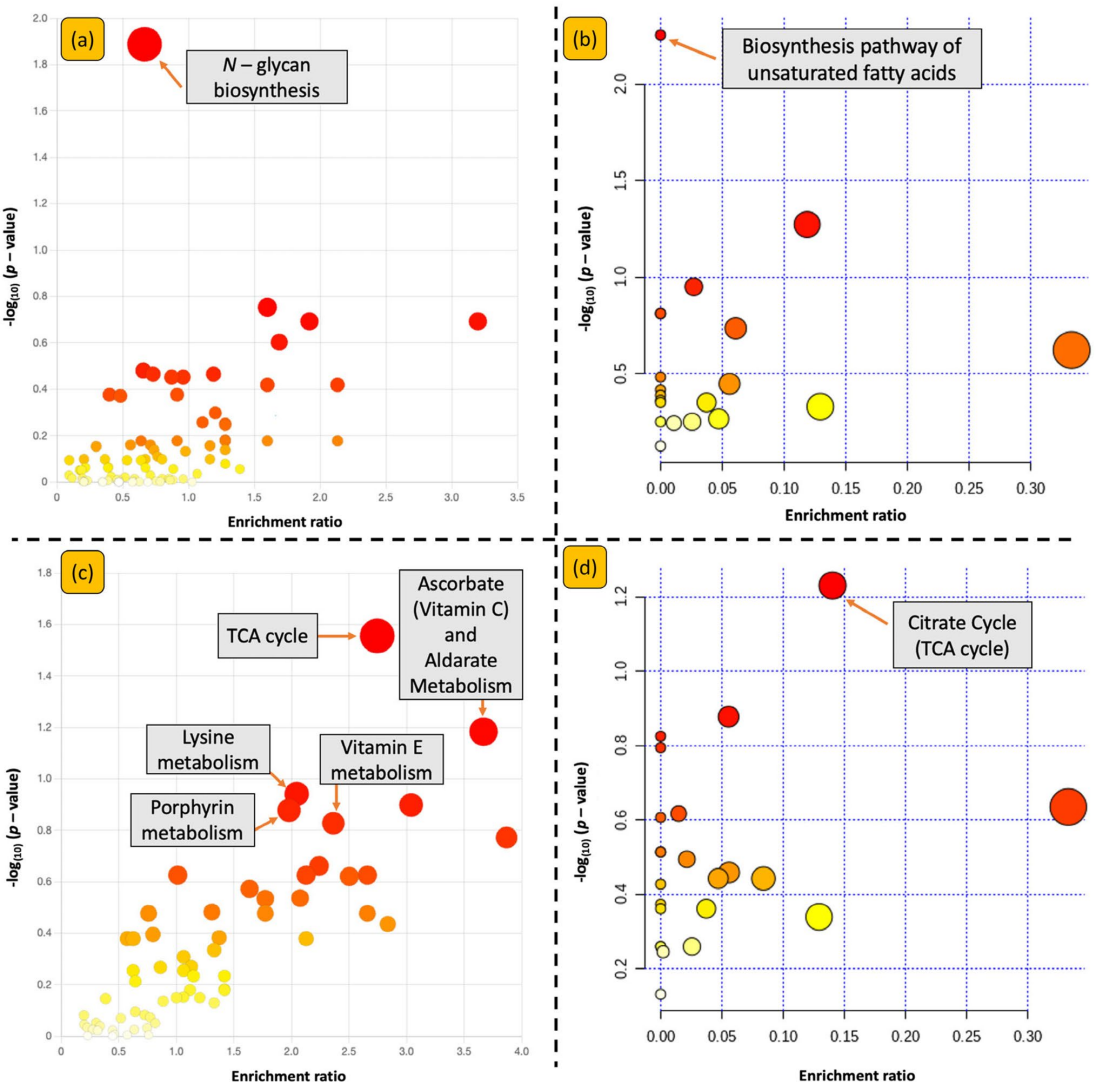
Pathway enrichment analysis results from MetaboAnalyst 4.0. This functional analysis was performed using all *m/z* features, their corresponding MW *U*-test (age) and KW-ANOVA scores (ethnicity), and *p*-values. Subfigures (**a**) and (**b**) represent ethnicity-specific pathways derived from LC-MS and GC-MS data, respectively. Subfigures (**c**) and (**d**) display age-specific pathways from LC-MS and GC-MS data, respectively. The y-axis represents the − log_(10)_ *p*-values from pathway enrichment analysis, with *p*-value threshold set at 0.05. The x-axis shows pathway enrichment expressed as the ratio of observed hits to expected hits on a pathway, where expected hits are based on chance alone

Pathway enrichment analysis was performed using the most important *m*/*z* features, determined by their VIP scores being higher than 1 from PLS-DA models used to predict age and ethnicity. Features selection returned 1574 features for LC–MS and 561 features for GC-MS with VIP scores > 1. Figure 4 shows the output in form of a bubble plot with each pathway represented with a circular node. For simpler interpretation, each filled circle corresponds to a metabolic pathway or a biological process, its size represents importance of that pathway in the context of analysis and its colour profile (yellow to red) infer the statistical significance (low to high) of these computed enrichments. This allowed us to prioritise selection of relevant enriched pathway for further interpretation, with the published literature. Typically, a large and red circle tends to be a pathway of high priority given its enrichment and significance. Pathways with significant hits have been highlighted. Note, not all the named pathways had statistically significant *p-*values, i.e., *p-*value below the set threshold value of 0.05 post Benjamini–Hochberg false discovery rate (BH-FDR) correction. Only those truly significant (i.e., FDR adjusted *p-*value ≤ 0.05) are discussed below.

## Discussion

Literature reviews reveal that the study of ethnic difference in men’s metabolic profiles has to date received very little attention. As noted by Vasishta et al. (2022), many metabolomics studies examining the metabolome from an ethnic perspective yield inconclusive result. This lack of clarity often stems from factors such as insufficient sample sizes, issues with metadata (e.g., gender, BMI, waist-to-hip ratio) either being mismatched or self-reported, or the constraints of limited case–control study designs (Hu et al., 2022). Our study follows recommendations from the community (Trivedi et al., 2017) and had 572 well-matched, fasted serum samples from community dwelling subjects with at least a 113 samples in each class for any single statistical analysis performed. These results are therefore a good representation of the ethnic phenotypes in the UK (White European, South Asian and African Caribbean) that have been characterised by metabolomics approaches. We were able to obtain metabolomic profiles of these men using LC–MS and GC–MS and could classify accurately these men using their metabolic signatures into ethnic phenotypes and age groups. To achieve this, we leveraged PLS-DA and validated this supervised learning method using bootstrapping and permutation testing. Finally, in this process the key contributing input variables (metabolites) were ranked and used for enriched metabolic pathway analysis using MetaboAnalyst. Below are discussions of the main pathways that we propose are biochemically distinct between ethnic and age phenotypes.

### *N*-Glycan biosynthesis in ethnicity

With Fisher’s exact test (FET) *p-*value of 0.02, *N-*glycan biosynthesis was the only highly significant impacted pathway by ethnicity specific discriminators (Fig. 4a). Gebrehiwot et al. (2018) defined the inter-ethnic variations of the *N*-glycome in serum in healthy individuals of US origin including South Indian, Japanese, and Ethiopian populations. These authors demonstrated an ethnicity-specific *N*-glycan expression pattern with 13 out of 51 glycans exclusively detected in Ethiopians. Immunoglobulin G (IgG), the most abundant circulating immunoglobulin, is heavily glycosylated, the alteration of which imposes a dysregulated immune-inflammatory response leading to increased risk of cardiovascular disease development. IgG-associated *N-*glycan traits are differentially associated with the development of hypertension in a population-pooled analysis of 4757 participants of different ethnicities (Chinese Han, Croatian Korcula, Croatian Vis and Scottish Orkney) (Wang et al., 2016). In a similar study, at least 10 IgG *N*-glycan traits associated with incident hypertension in ethnic minorities of China (namely, Uygur, Kazak, Kirgiz, and Tajik) (Liu et al., 2018). Although previous studies have not established a clear link between stratified ethnicity and IgG *N*-glycosylation, *N*-glycan traits show differential correlations with ethnic groups. This information can be further investigated in future population-based studies across both genders to gain a clearer understanding of how *N*-glycans vary across ethnicities and whether these variations are significant.

### Biosynthesis of unsaturated fatty acids and ethnicity

Ethnicity specific metabolic conversion of fatty acids such as linoleic acid (LA) and arachidonic acid (AA) have been reported previously (Abdelmagid et al., 2015). Recently, a study performed in a US based cohort identified 259 metabolites including *γ*-LA, an intermediate between LA and AA in the conversion chain to be differentiating Black and White women based on their ethnicity. The study linked these metabolites to the increased susceptibility of coronary heart disease observed in these women (Hu et al., 2022). Such changes can be attributed to ethnicity specific genetic variations for enzymes involved in long-chain fatty acid synthesis, such as delta 5-desaturase (D5D) and delta 6-desaturase. Polymorphisms of genes encoding these enzymes have been reported to alter in Caucasian and Asian adults, resulting in differential plasma fatty acid profiles (Merino et al., 2011). Omega-3 polyunsaturated fatty acids suggest that ethnic diversity affects cardiovascular outcomes through variations in lipid profiles and glucose homeostasis (Patel et al., 2010). Ethnic differences are also associated with distinct inflammatory phenotypes; for instance, several studies indicate that Black individuals have higher levels of inflammatory biomarkers compared to Hispanics, Whites, or Asians (Abdelmagid et al., 2015; Carroll et al., 2009; Stowe et al., 2010). Anti-inflammatory long-chain polyunsaturated fatty acids (LC-PUFAs) are often prescribed to mitigate sub-clinical inflammation associated with aging. A question remains as to whether dysregulation or heterogenicity of fatty acid metabolism biologically contributes to ethnicity-based differences in health outcomes, considering the age of the cohort. Whether this contribution is independent of other factors should also be investigated. However, to definitively address these questions, it is necessary to investigate clinical measurements of known disease precursors and hormonal data. Previous research (Eendebak et al., 2017) has shown that these factors have differential impacts by ethnicity in men from the same cohort as ours.

Despite being classified as an unmodifiable risk factor for poor cardiovascular outcomes, ethnicity is increasingly recognised to have a significant impact on patient outcomes. For instance, African-Caribbeans, Hispanics, and other ethnic minorities have a substantially increased prevalence of hypertension (HTN) and type 2 diabetes mellitus, which automatically imposes a higher baseline threshold for developing end-organ damage, including heart failure (HF) (Kim et al., 2010). In this cohort Spearman’s correlation of the clinical and biochemical metadata (Fig. S8) revealed no strong correlation of age, clinical characteristics, morbidity, and physiology with ethnicity. There was a moderately strong negative correlation between ethnicity and age (*r* = − 0.47). There was a weak negative correlation observed between ethnicity and waist-to-hip ratio (*r* = − 0.31) and shbg (*r* = − 0.15). Age showed moderate positive correlation to shbg (*r* = 0.36), high blood pressure (*r* = 0.35) and stroke (*r* = 0.22) all of which are expected to vary with age. As such, in absence of any strong correlation there is very little indication of any significant confounding effect from comorbidities and lifestyle present in this cohort. It should be noted that association with age related ailments such as high blood pressure and heart condition or stroke is expected in a cohort consisting of middle aged and older men. An essential takeaway for epidemiological metabolomics analysts would be the strengthened requirement to account for confounding effects of both age and ethnicity in future human metabolic profiling studies using serum.

### Citrate (TCA) cycle and ageing

In the present study citrate, as an indicator of the TCA cycle, was significantly impacted (FET *p*-value = 0.04) by age specific classifiers from our discriminant analysis. This finding that citric acid was upregulated in older age groups corroborates findings from a large-scale UK based phenotyping study in serum by Dunn et al. (2015). Moreover, Saito et al. (2016) also claim energy metabolites of citrate cycle to be significantly different between young and old men. Whilst the TCA cycle has been linked to ageing and age-associated diseases (Chin et al., 2014) it has not been co-studied for influence from ethnicity of individuals. To determine if there was a confounding effect of ethnicity on citric acid expression in our cohort, box whiskers plots and pairwise *t-*test was performed which showed insignificant difference in citrate level across ethnicities (Fig. S9). Extracellular citrate accumulation in the senescent cells is considered to be a marker for chronological age independent of sex, BMI or telomere length (Mycielska et al., 2022) and abnormalities in the citrate cycle have been associated to declined intrinsic capacity in older individuals (Pan et al., 2022). In rodents (Chen et al., 2018), and in humans, citrate stored in bones is released into extracellular matrix in osteoporosis, a disease more prevalent in the later age.

The citrate cycle occurs within the mitochondria and involves a series of chemical reactions resulting in the production of diverse metabolites mediating several cellular functions, including epigenetic modifications, post-translational modifications, and diverse biosynthetic pathways (Martínez-Reyes & Chandel, 2020). There is growing evidence that links TCA metabolic dysregulation with ageing and age-associated diseases. A UK-based phenotyping study in a large populations (*n* = 1200) found citric acid is not gender-specific and increased with age in both males and females (Dunn et al., 2015) where ethnicity was not accounted for.

### Porphyrin metabolism and other age-related pathways

The impact on porphyrin metabolism was noticeable with respect to age. There is not much information available to link porphyrin metabolism to age. Reports of alcohol consumption causing disturbances to porphyrin metabolism have been published (Doss et al., 2000). Alcohol is considered a porphyrinogenic agent and a cause for hepatic coproporphyrinuria later manifested as alcohol-induced liver damage. Among our subjects 13/572 report chronic liver-related disease or dysfunction. It is unlikely for this enrichment to have been contributed from these 13 men; this association between age and porphyrin metabolism should be further investigated. To investigate whether alcohol intake in this population influenced ageing in this population, we performed mummichog analysis on two subsets of our population i.e., drinkers and non-drinkers. Porphyrin metabolism pathway remained impacted (Fig. S10a, b) in both cases suggesting the alcohol intake in these subjects did not play a role in its enrichment.

Anthranilic acid, a metabolite produced in the L-tryptophan-kynurenine pathway, was observed to increase with age (Fig. S6). Previous studies report L-tryptophan-kynurenine pathway associated with ageing and age-related diseases. Dunn et al. (2015) reported decreasing tryptophan with male ageing which can be explained by increased muscle mass turnover. An increase in kynurenine causes tryptophan to decrease and this has been linked with age-specific cognitive decline (van der Goot & Nollen, 2013). Kynurenine is a downstream metabolite of tryptophan metabolism and a substrate for formation of anthranilic acid (Ramos-Chávez et al., 2018). Therefore, decrease in tryptophan can explain increasing anthranilic acid with progressing age. A recent study linked metabolic biomarkers of decreased intrinsic capacity in elderly to tryptophan metabolism among other key pathways reported (Pan et al., 2022).

We note that some of the metabolites (Figs. S6 and S7) may not be of endogenous origin yet differentially expressed. Metabolites like gallic acid (Kahkeshani et al., 2019) may be from anti-inflammatory, neuropsychological and metabolic supplements, caffeic acid from coffee intake (Bastianini et al., 2018) whereas potential metabolites from use of cosmetic and anti-ageing solutions such as L-rhamnose (Pageon et al., 2019) were also detected. We found kaempferol—a polyphenol administered as supportive treatment for diseases in some traditional medicinal approaches (Ren et al., 2019). This could be indicative of the cultural diversity within this population and their self-care habits. Detection of such metabolites is expected alongside endogenous metabolites in a global profiling experiment performed using highly sensitive, chemically specific MS-platforms.

### Strengths, limitations, and outlook

There are some limitations to this study. Moreover, the same ethnic groups in different countries may have different serum metabolic profiles. These differences in serum metabolomes could be attributed to many external factors such as diet, environment, socio economic factors and access to healthcare in early age (Goodacre, 2007). Additional metadata including dietary intake, medication, significant lifestyle factors and any exposome influencers would enhance findings in such population-based metabolomics studies. Ethnicity in this population was determined using ethnic origins of one or both parent/s going back up to four generations. As robust as this method is, it is not a universal method to determine or record ethnicity in populations worldwide. Studies relying on any self-reported ethnicity data for e.g., Navarro et al. (2023) or ethnicity otherwise estimated or recorded, may not always be accurate or reliable. Therefore, the phenotype associated to such data may not be confidently defined; that is to say, there may not be a strong difference for these different groups. That said this is a large study where relatively large populations of individuals were measured: 287 White Europeans, 143 South Asians and 142 African Caribbeans.

Moreover, we address the inter-individual heterogeneity within our cohort, with respect to demographic data, different clinical parameters, and comorbidities. The study is not intended to identify metabolic biomarkers of certain disease entities, but rather to illustrate the global association of metabolic profiles with aging and ethnicity. We did not therefore attempt to investigate whether ethnicity is directly or independently related to the identified metabolites or metabolic pathways.

Our study highlights the potential of metabolomics in routine clinical decision making, where currently despite a suite of administrable therapies available in hospitals for some cases, the decision-making is often a struggle due to poorly understood patient response owing to population heterogeneity (Caiazzo et al., 2022). Koeken et al. (2022) sets the premise to show how metabolomic profiles can be used to predict immune responses which in turn can enable delivery of effective and tailored vaccines and therapies in populations. We encourage further work in this area with added analytical platforms, inclusion of other genders, wider ethnic diversity, larger sample size, additional metadata, and a multi-center validation mechanism to accelerate research in healthy ageing to alleviate socio-economic burdens. This could pave way for personalised biomarkers in future geared towards predictive, diagnostic, and therapeutic interventions needed for inclusive and healthy ageing.

## Conclusion

This work is our contribution towards the UN’s ‘decade of healthy ageing’ declaration. With the novel ethnicity specific metabolome changes reported here, we are a step closer to understanding differential mechanisms of ageing across these ethnic groups. We report that men of White European, South Asian, and African Caribbean origin have distinct metabolic signatures in serum with prediction potential of their ethnicity and age group. We report changes in energy and fatty acid metabolism driven by age and ethnicity of these men. This approach will need to be adapted to a wider range of ethnicities both within the UK and globally. Future work should include other genders, wider ethnic diversity, larger cohorts, longitudinal data, and multi-centre validation to accelerate research in healthy ageing.

## Supplementary Information

Below is the link to the electronic supplementary material.

Supplementary file 1 (DOCX 7058 KB)

## Data Availability

All data files are available in .*mzXML* format at MetaboLights repository (Yurekten et al., 2024) at https://www.ebi.ac.uk/metabolights/editor/www.ebi.ac.uk/metabolights/MTBLS10303. The codes used for spectral pre-processing, auto-correction, normalisation, PCA, and PLS-DA are available at https://github.com/BioSpec/cluster-toolbox-v2.0.
