# Correction: Metabolomic heterogeneity of ageing with ethnic diversity: a step closer to healthy ageing

**DOI:** 10.1007/s11306-025-02247-x

**Published:** 2025-04-01

**Authors:** Dakshat Trivedi, Katherine A. Hollywood, Yun Xu, Fredrick C. W. Wu, Drupad K. Trivedi, Royston Goodacre

**Affiliations:** 1 Centre for Metabolomics Research (CMR), Department of Biochemistry, Cell, and Systems Biology, Institute of Systems Molecular and Integrative Biology, University of Liverpool, Liverpool, UK; 2 Clinical Metabolomics Unit (CMU), Human Development and Health, Institute of Developmental Sciences, University of Southampton, Southampton, UK; 3 Manchester Institute of Biotechnology (MIB), School of Chemistry, University of Manchester, Manchester, UK; 4 Andrology Research Unit (ARU), Division of Endocrinology, Diabetes and Gastroenterology, School of Medical Sciences, Faculty of Biology, Medicine and Health, University of Manchester, Central Manchester University Hospitals NHS Foundation Trust, Manchester, UK

**Correction to Metabolomics:** 10.1007/s11306-024-02199-8

In this article, the conflict of interest section has been updated with the sentence “Prof. Royston Goodacre is the editor-in-chief of Metabolomics”. And it should have been “All authors declare no conflict of interest. Prof. Royston Goodacre is the editor-in-chief of Metabolomics.”

The original article has been corrected.

