# Supplementary material for: Metabolomic heterogeneity of ageing with ethnic diversity: a step closer to healthy ageing
Source: Metabolomics. 2024 Dec 15;21(1):9. doi: 10.1007/s11306-024-02199-8 (PMC11646956; doi:10.1007/s11306-024-02199-8)
Supplement: Supplementary file 1 — (DOCX 7058 KB) [file 11306_2024_2199_MOESM1_ESM.docx]

**Metabolomic heterogeneity of ageing with diversity – a step closer to healthy ageing**

**Dakshat Trivedi, Katherine A. Hollywood, Yun Xu, Fredrick C.W. Wu, Drupad K. Trivedi** **and** **Royston Goodacre**

**
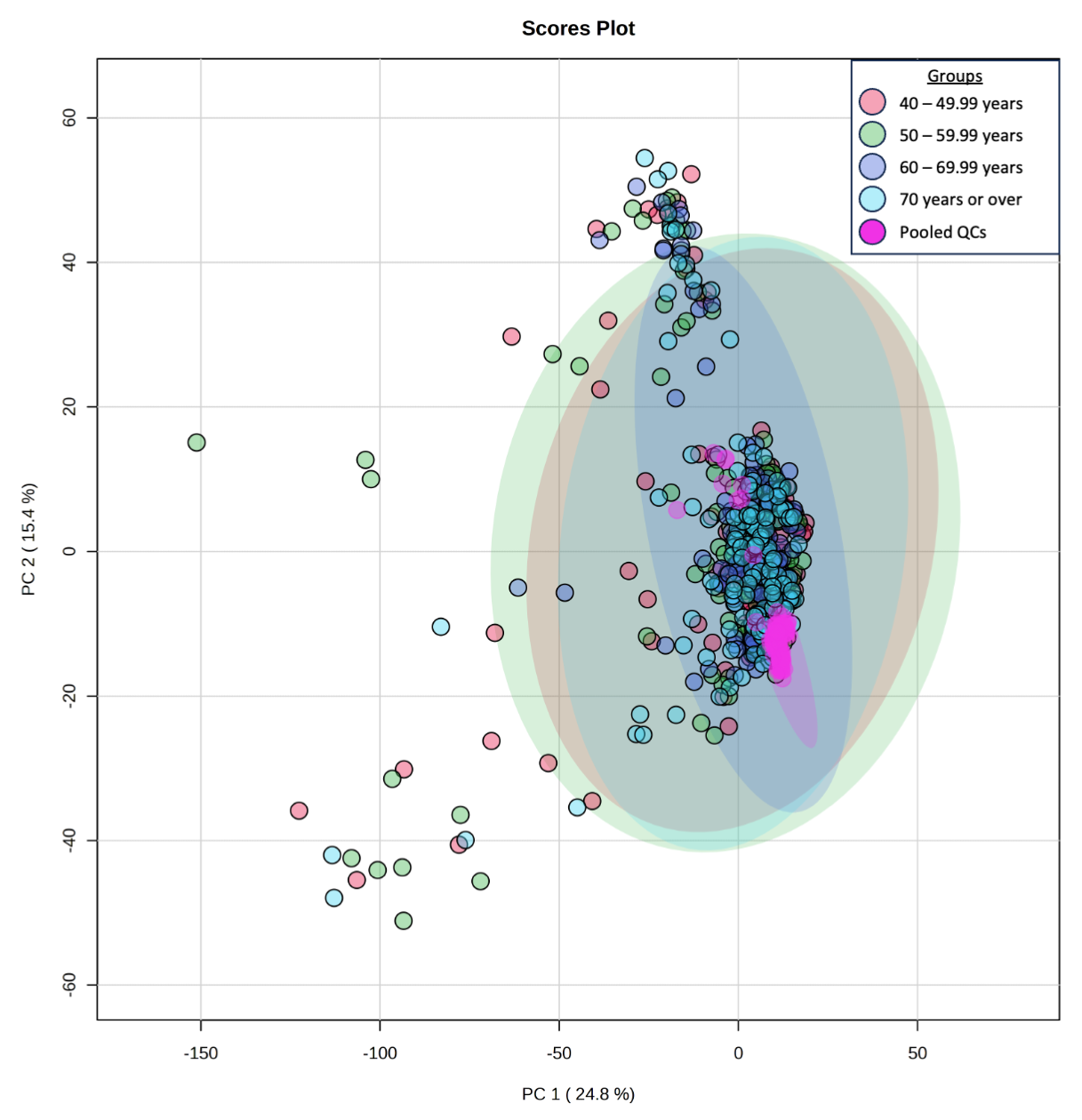

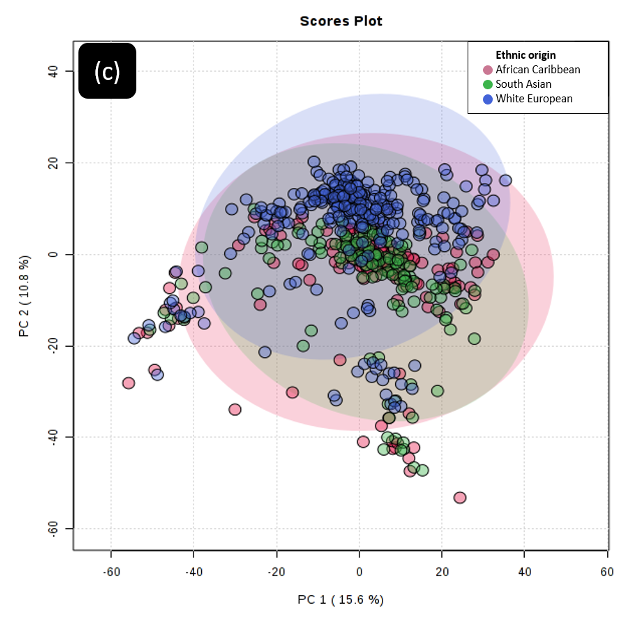
**

(d)


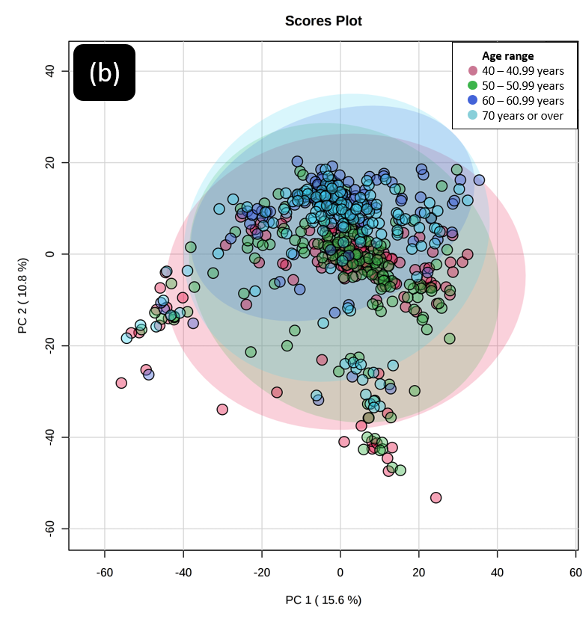

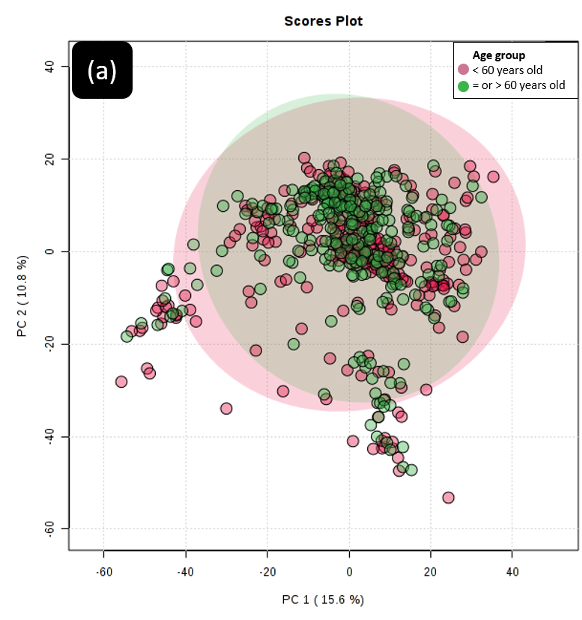


**Figure S1** PCA scores plots showing the lack of clustering of data by groups or separation patterns within the subject data. These are the same scores plots but are visualised differently: (a) four groups of age (40-49.99, 50-59.99, 60-69.99, ≥ 70 years); (b) two groups of those younger than 60 years and those equal to or above 60 years, (c) three groups based on their ethnic origin, i.e., African Caribbean, South Asian and White European and (d) all samples grouped in 4 age groups with QC samples included as a separate group. The X-axis of the scores plot shows total explained variance (TEV) of 15.6% (a,b,c) and 24.8% (d) along principal component 1 (PC1) and the Y-axis of the scores plot shows TEV of 10.8% (a,b,c) and 15.4% (d) along principal component 2 (PC2). In this unsupervised analysis, no *a priori* information about these different groups is provided for the statistical output.


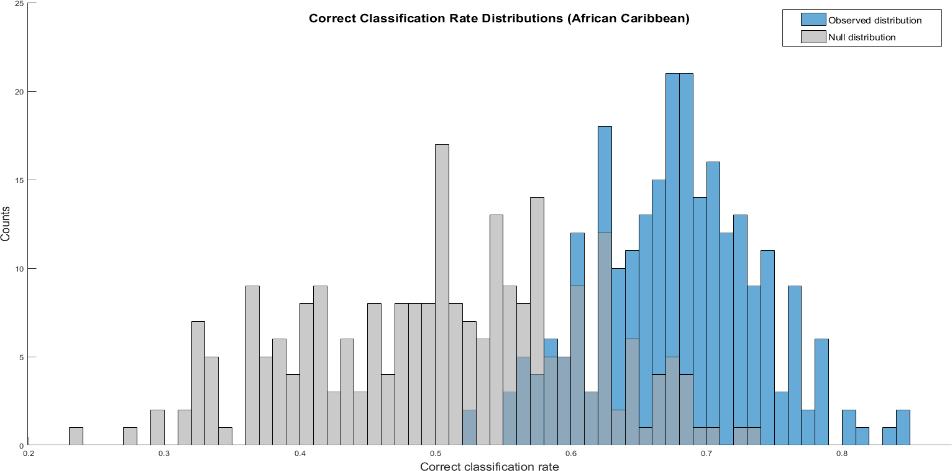

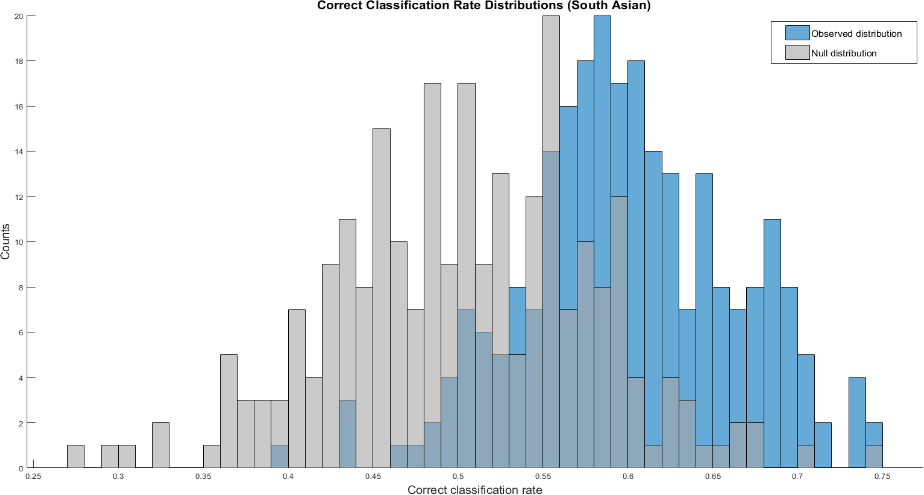

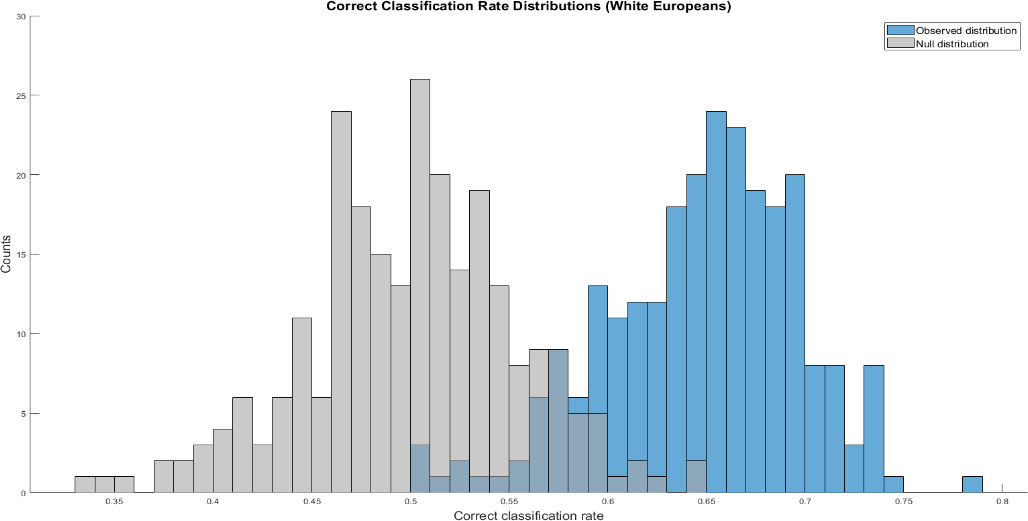


**Figure S2** The test set outputs of PLS-DA models using SMOTE for classification of age-adjusted for ethnicities for (a) South Asian, (b) White European and (c) African Caribbean. These histograms show the test set classification rates for 1000 PLS-DA bootstrap models (blue) with the null distribution from permutation testing (grey). SMOTE was employed to account for sample number imbalances.


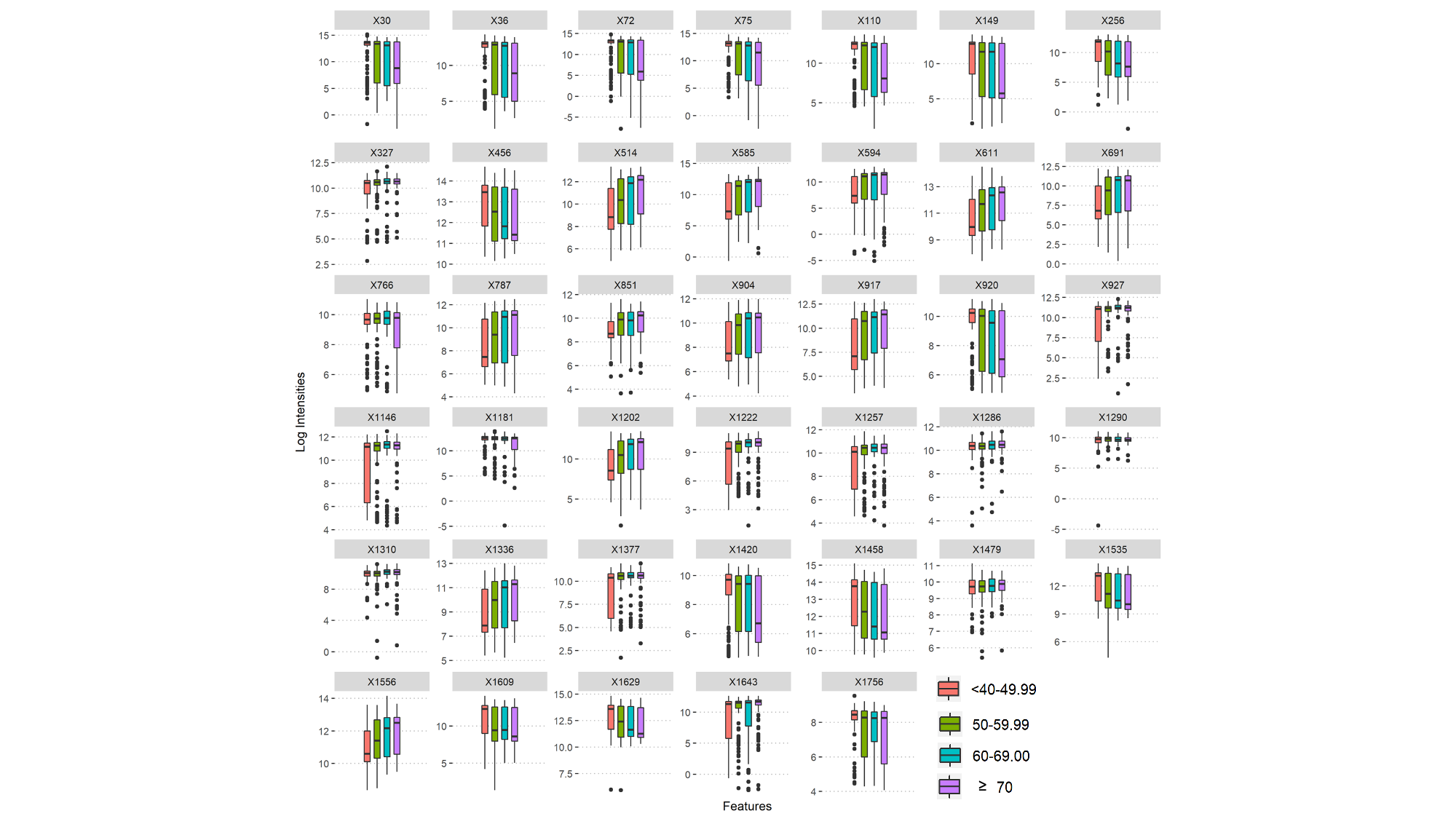


**Figure S3** Box whisker plots for the top 40 (top 10%) compounds that were discriminatory for four age ranges: 40-49.99, 50-59.00, 60-69.99 and ≥ 70 years of age. The y-axis in each plot corresponds to the natural log intensity values and x-axis denotes the feature. Corresponding putative annotations can be found listed in Table S1.


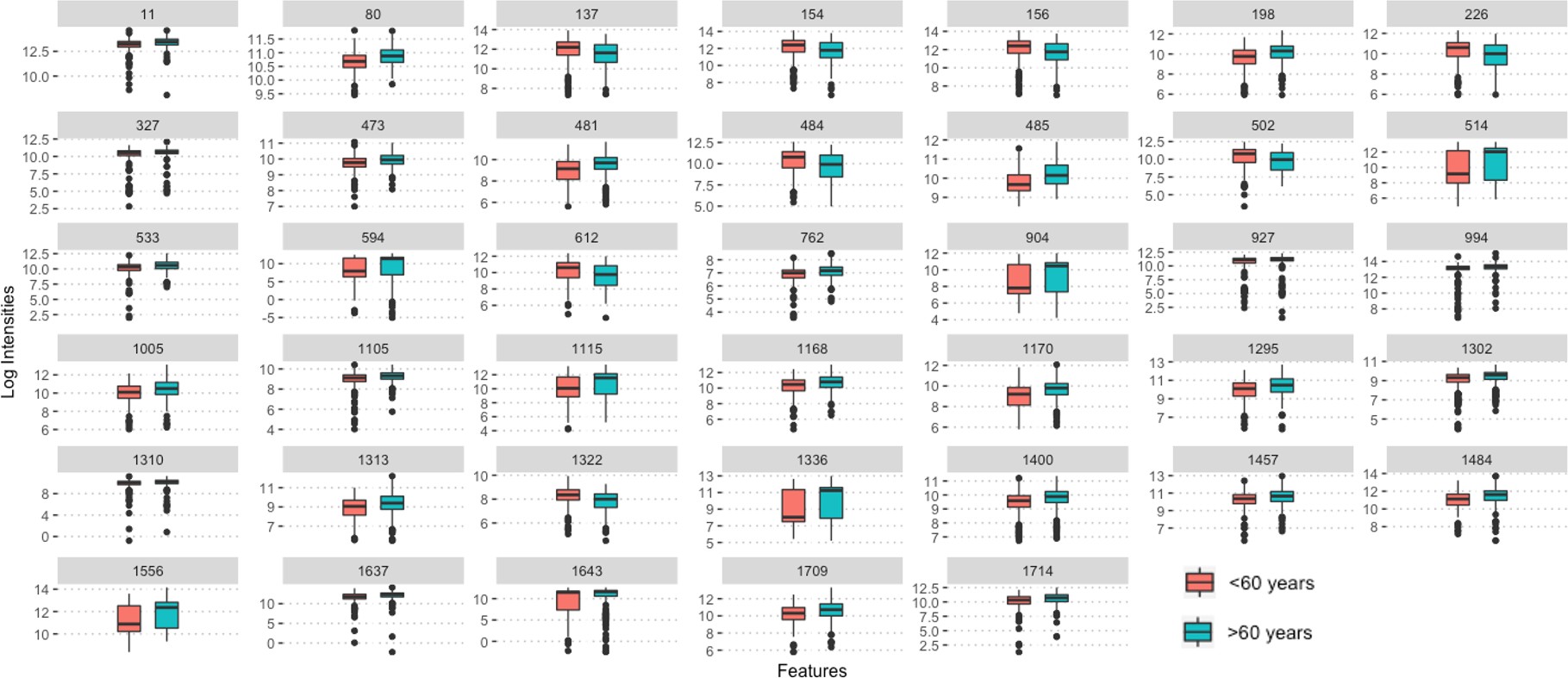

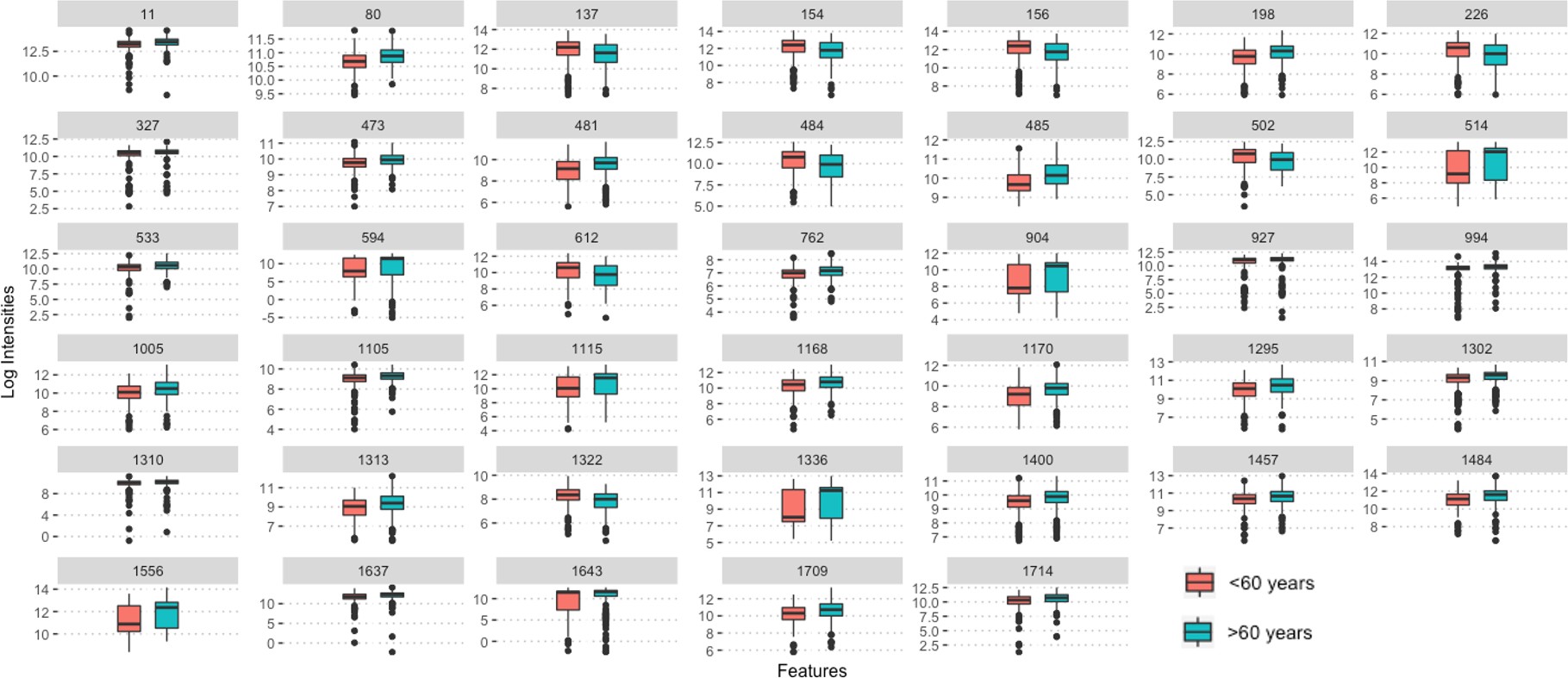

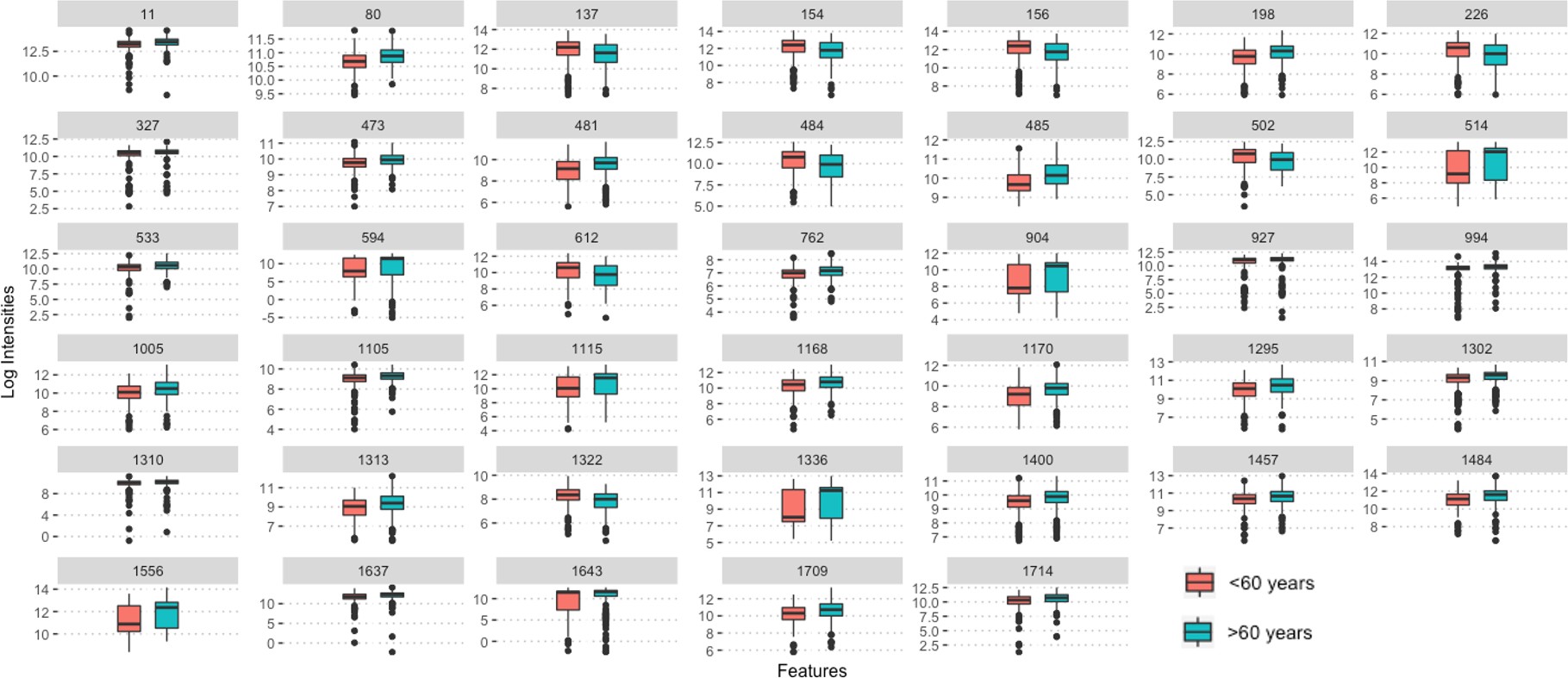


**Figure S4** Box whisker plots for all the top 40 (top 10%) that were discriminatory for two categories of age: < 60 and ≥ 60 years of age. The y-axis in each plot corresponds to the natural log intensity values and x-axis denotes the feature. Corresponding annotations can be found listed in Table S1.


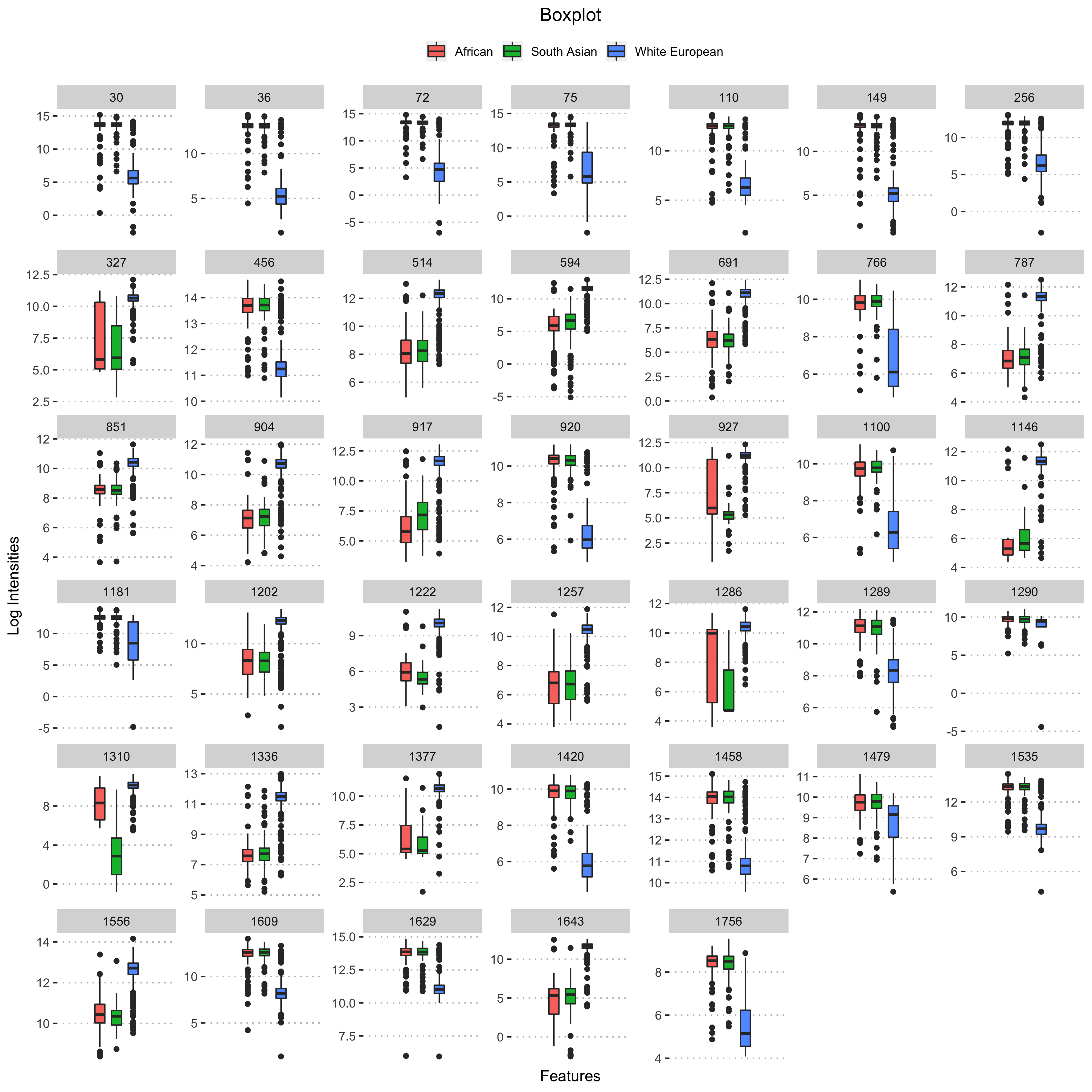


**Figure S5** Box whisker plots for all the top 40 (top 10%) for discriminating the three categories of ethnicity: African Caribbean, South Asian and White European. The y-axis in each plot corresponds to the natural log intensity values and x-axis denotes the feature. Corresponding putative annotations can be found listed in Table S2.


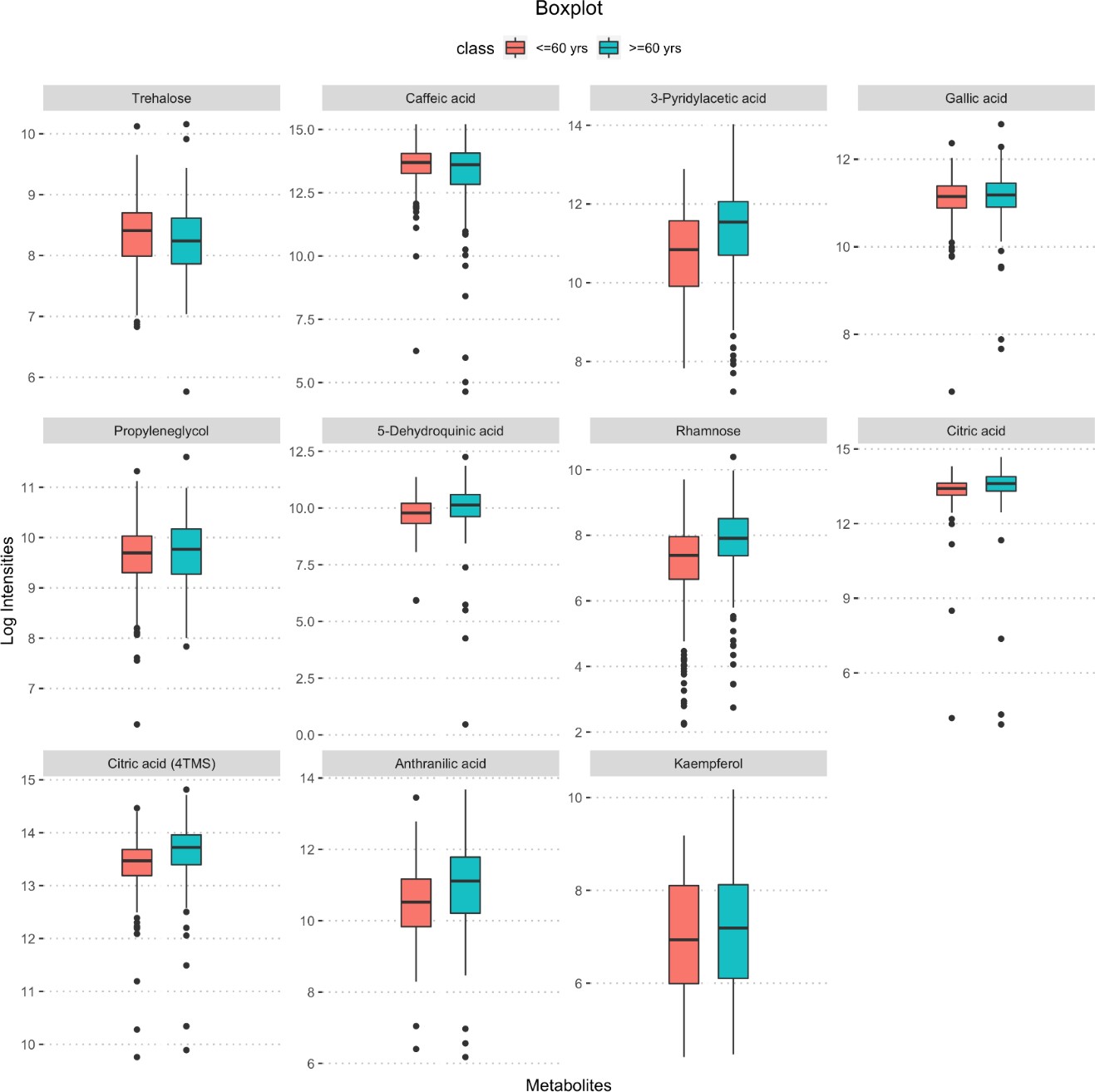


**Figure S6** Box whiskers plot for key metabolic features separating < 60 from ≥ 60 years, selected using VIP scores from PLS-DA.


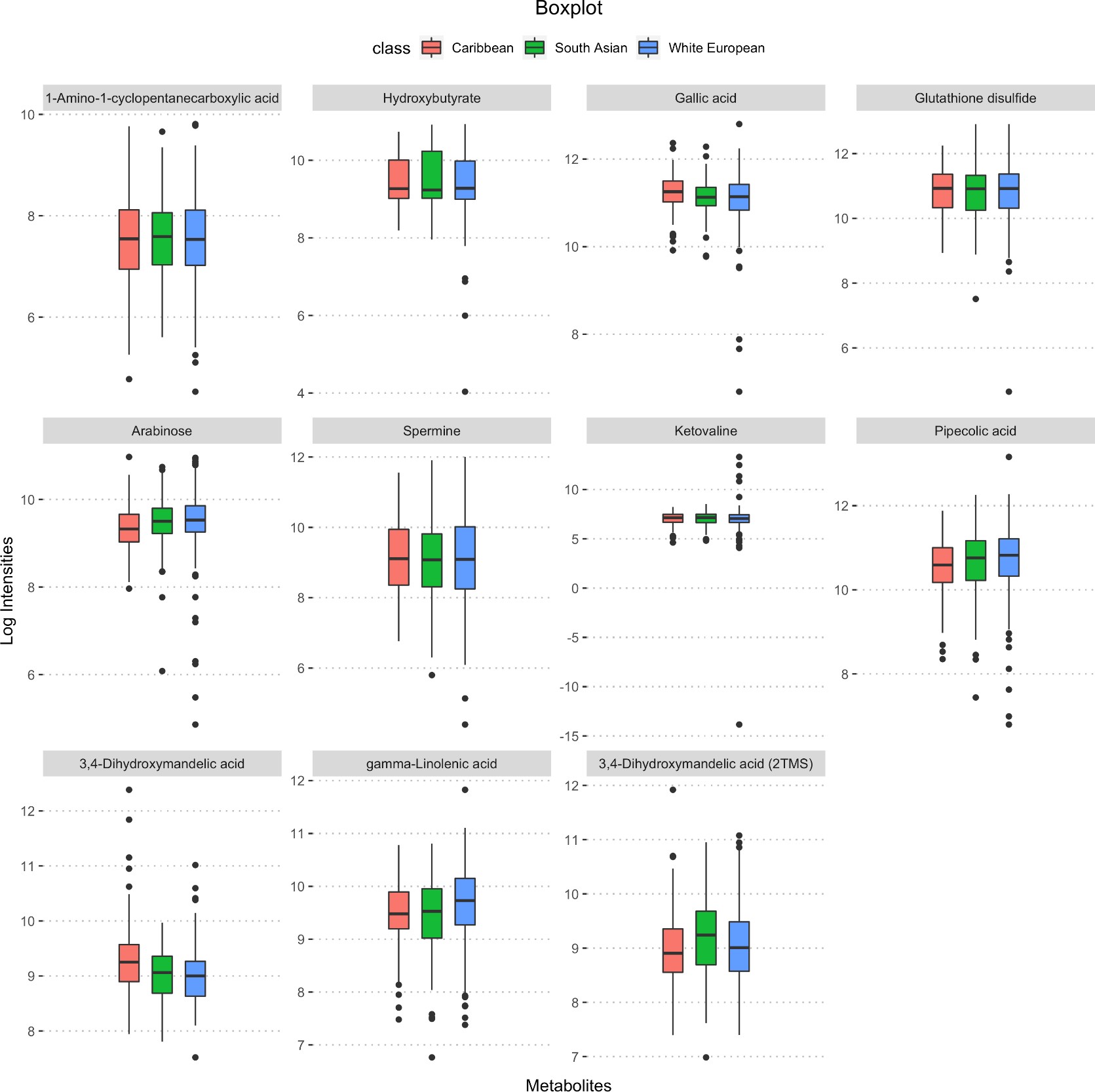


**Figure S7** Box whiskers plot for key metabolic features from ethnic origin-based selected using VIP scores from PLS-DA.

| **Sample descriptions** | **Number (n) of samples** |
| --- | --- |
| **Total serum samples (HUSERMET +EMAS)** | **709** |
| ‘Other’ ethnicity (specifics unknown) | 35 |
| Missing metadata (age, BMI etc.) | 33 |
| Insufficient sample volume | 69 |
| **Total number of samples not analysed** | **137** |
| **Total number of samples analysed (this study)** | **572** |
| White Europeans | 287 |
| South Asians | 143 |
| African Caribbeans | 142 |

**Table S1** Sample breakdown of the 709 serum samples available collectively from HUSERMET and EMAS cohorts of which a subset was analysed in this study due to exclusions explained below.

**Table S2** Putative identification of all metabolites from LC-MS analysis with VIP >1 with peak IDs.

| ***m*/*z*** | **Retention time** | **Tentative ID** | **Chemical formula** | **Adduct** | **Δppm** | **Peak**  **ID** |
| --- | --- | --- | --- | --- | --- | --- |
| 275.13702 | 7.3184 | Carboxylic acid (class) | C_27_H_42_O_10_ | [M+H+Na]2+ | 1 | 1290 |
| 318.19574 | 7.1989 | Methysergide | C_21_H_27_N_3_O_2_ | [M+H-2H_2_O]+ | 6 | 30 |
| 327.20098 | 6.4840 | Heptaethylene glycol | C_14_H_30_O_8_ | [M+H]+ | 1 | 256 |
| 333.18804 | 5.3492 | Citrulline | C_6_H_13_N_3_O_3_ | [2M+H- H_2_O]+ | 2 | 904 |
| 339.23729 | 6.3220 | (-)-Folicanthine | C_24_H_30_N_4_ | [M+H-2 H_2_O]+ | 9 | 851 |
| 347.20365 | 5.8465 | (S1)-Methoxy-3-heptanethiol | C_8_H_18_OS | [2M+Na]+ | 4 | 1336 |
| 371.22709 | 6.6301 | Phosphatidylcholine | C_41_H_67_O_8_P | [M+H+Na]+ | 2 | 110 |
| 382.25557 | 7.7591 | 2-Hydroxylauroylcarnitine | C_19_H_37_NO_5_ | [M+Na]+ | 2 | 1222 |
| 383.26346 | 6.5151 | Narasin | C_43_H_72_O_11_ | [M+2H]+ | 6 | 327 |
| 389.21408 | 6.4840 | 1,2-Di-O-myristoyl-3-O-(6-sulfoquinovopyranosyl) glycerol | C_37_H_70_O_12_S | [M+H]+ | 1 | 1181 |
| 391.22979 | 6.0399 | Oxatomide | C_27_H_30_N_4_O | [M+H-2 H_2_O]+ | 1 | 787 |
| 396.27112 | 8.2456 | 3-hydroxytridecanoyl carnitine | C_20_H_39_NO_5_ | [M+Na]+ | 2 | 1310 |
| 405.75935 | 5.7002 | Phosphatidylethanolamine | C_47_H_72_NO_8_P | [M+2H]+ | 6 | 920 |
| 410.28675 | 8.8908 | 2-Hydroxymyristoylcarnitine | C_21_H_41_NO_5_ | [M+Na]+ | 2 | 1643 |
| 415.25327 | 6.7606 | Phosphatidylglycerole (18:1(11Z)/16:0) | C_40_H_78_O_13_P_2_ | [M+2H]+ | 3 | 149 |
| 435.25594 | 6.2179 | Withanolide A | C_28_H_38_O_6_ | [M+H-2 H_2_O]+ | 4 | 1377 |
| 437.23535 | 5.1962 | Pectenotoxin 3 | C_47_H_68_O_15_ | [M+2H]+ | 2 | 456 |
| 439.30759 | 9.0716 | Phosphatidylcholine (24:1(15Z)/22:2(13Z,16Z)) | C_49_H_91_O_8_P | [M+H]+ | 3 | 611 |
| 440.29726 | 8.2924 | Phosphatidic acid 24:1(15Z)/22:4(7Z,10Z,13Z,16Z) | C_49_H_87_O_8_P | [M+2Na]+ | 3 | 1286 |
| 446.3155 | 9.2605 | alpha-Tocotrienoxyl radical | C_29_H_43_O_2_ | [M+Na]+ | 3 | 1556 |
| 449.27157 | 6.6509 | Seryllysine | C_9_H_19_N_3_O_4_ | [2M+H- H_2_O]+ | 1 | 1146 |
| 459.27948 | 6.8651 | PC(MonoMe(11,5)/DiMe(11,3)) | C_49_H_87_NO_10_P | [M+H]+ | 4 | 36 |
| 461.3078 | 8.4008 | Carboxylic acid (class) | C_28_H_46_O_4_S | [M+H- H_2_O]+ | 3 | 917 |
| 479.2819 | 6.6509 | Carboxylic acid (class) | C_27_H_42_O_5_S | [M+H]+ | 1 | 1257 |
| 481.26155 | 5.3861 | Cavipetin E isomer 2 | C_29_H_40_O_8_ | [M+H-2 H_2_O]+ | 4 | 1629 |
| 483.33373 | 9.0562 | Frangulanine | C_28_H_44_N_4_O_4_ | [M+H- H_2_O]+ | 1 | 585 |
| 491.31819 | 8.1007 | Carboxylic acid (class) | C_29_H_46_O_4_S | [M+H]+ | 2 | 691 |
| 493.29767 | 6.7815 | Carboxylic acid (class) | C_28_H_44_O_5_S | [M+H]+ | 1 | 927 |
| 504.33748 | 5.3861 | Carboxylic acid (class) | C_21_H_19_I_4_NO_11_ | [M+H]+ | 9 | 766 |
| 507.31359 | 7.1365 | Carboxylic acid (class) | C_29_H_46_O_5_S | [M+H]+ | 1 | 594 |
| 525.28766 | 5.5531 | Chalciporone | C_16_H_21_NO | [M+H]+ | 2 | 1458 |
| 547.3319 | 7.0425 | CE(5:0) | C_32_H_54_O_2_ | [M+H]+ | 1 | 72 |
| 548.36348 | 5.5531 | Tetracosatetraenoyl carnitine | C_31_H_53_NO_4_ | [M+2Na-H]+ | 9 | 1479 |
| 591.35802 | 7.1261 | Melanostatin | C_13_H_24_N_4_O_3_ | [M+H]+ | 1 | 75 |

**Table S3** Putative identification of the five most significant compounds (LC-MS) among the top ten per cent discriminating features with VIP > 1 in age model 1 (40-49.99, 50-59.00, 60-69.99 and ≥ 70 years of age).

| **m/z** | Retention Time | Putative ID  (HMDB) | MSI  level | Chemical Formula | Class/sub-class | Adduct | ∆ppm |
| --- | --- | --- | --- | --- | --- | --- | --- |
| 383.26346 | 6.5151 | 11'- Carboxy-g-tocotrienol | 3 | C_25_H_36_O_4_ | Lipids/Prenyl Lipids | [M+H- H_2_O]+ | 13 |
| 410.28675 | 8.8908 | 2- Hydroxy  -myristoylcarnitine | 3 | C_21_H_41_NO_5_ | Fatty acyls/fatty acid esters | [M+Na]+ | 2 |
| 449.27157 | 6.6509 | Organooxygen  metabolite | 3 | C_33_H_48_N_2_O_11_S | Organooxygen/  Carbohydrate | [M+Na]+ | 4 |
| 461.32081 | 9.0665 | 27-Nor-pentol | 3 | C_26_H_46_O_5_ | Steroid/Bile acid | [M+Na]+ | 6 |
| 493.29767 | 6.7815 | 3-Hydroxy-dodecanedioate | 3 | C_12_H_22_O_5_ | Hydroxy acids/medium-chain hydroxy acid | [M+H]+ | 1 |
| 507.31358 | 7.1365 | Isoflavonoid  metabolite | 3 | C_21_H_22_O_5_ | Isoflavonoid/  Furanoisoflavonoids | [M+H]+ | 1 |

**Table S4** Putative identification of the five most significant compounds (LC-MS) among the top ten per cent discriminating features with VIP > 1 in age model 2 (< 60 from ≥ 60 years).

| m/z | Retention Time |  | Putative ID  (HMDB) | MSI  level | Chemical Formula | Class/sub-class | Adduct | ∆ppm |
| --- | --- | --- | --- | --- | --- | --- | --- | --- |
| 129.01806 | 1.8487 |  | 5-Hydroxy-2-furoic acid | 3 | C_5_H_4_O_4_ | Furans/Furoic acids | [M+H]+ | 1 |
| 168.06277 | 0.6876 |  | 3-Keto-5-aminohexanoic acid | 3 | C_6_H_11_NO_3_ | Keto-acid/Medium-chain keto-acid | [M+Na]+ | 2 |
| 219.04689 | 3.8731 |  | Hypotaurine | 3 | C_2_H_7_NO_2_S | Sulfinic acid | [2M+H]+ | 1 |
| 255.21000 | 8.0178 |  | Dihydrotestosterone | 3 | C_19_H_30_O_2_ | Steroids/Androstane steroid | [M+H-2 H_2_O]+ | 6 |
| 309.15000 | 10.1210 |  | 2-Hydroxyestrone | 3 | C_18_H_22_O_3_ | Steroids/Estrane steroids | [M+Na]+ | 15 |

**Table S5** Putative identification of the five most significant compounds (LC-MS) among the top ten per cent discriminating features with VIP > 1 in ethnicity model (African Caribbean, South Asian and White European).

| m/z | Retention  Time | Putative ID  (HMDB) | MSI  level | Chemical Formula | Class/sub-class | Adduct | ∆ppm |
| --- | --- | --- | --- | --- | --- | --- | --- |
| 327.20097 | 6.4840 | Cortisol | 3 | C_21_H_30_O_5_ | Steroids/  Hydroxy steroids | [M+H-2 H_2_O]+ | 13 |
| 461.32340 | 10.2977 | 27-Nor-pentol | 3 | C_26_H_46_O_5_ | Steroid/Bile acid | [M+Na]+ | 6 |
| 481.26155 | 5.3861 | Butenyl carnitine | 3 | C_11_H_19_NO_4_ | Fatty acyls/Fatty acid esters | [2M+Na]+ | 20 |
| 525.28765 | 5.5531 | 2aryl-benzofuran -flavonoid metabolite | 3 | C_39_H_40_O_15_ | 2-Arylbenzofuran Flavonoids | [M+H-2 H_2_O]+ | 3 |
| 547.33189 | 7.0425 | Isoflavonoid metabolite | 3 | C_27_H_28_O_12_ | Isoflavonoid/  Furanoisoflavonoids | [M+H]+ | 4 |


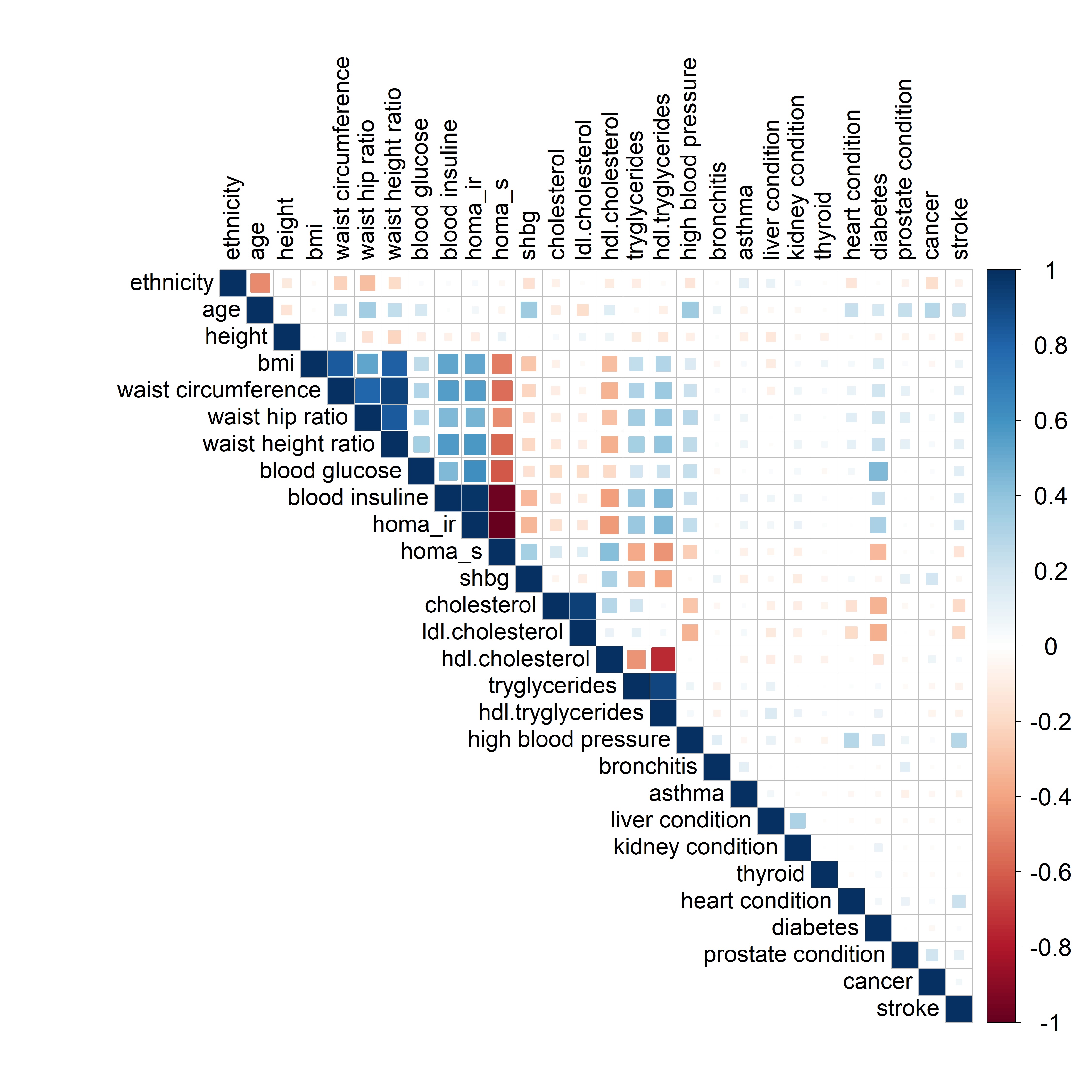


**Figure S8** Spearman’s correlation matrix of available metadata with ethnicity and age. The colour bar shows the correlation coefficient (*r*).


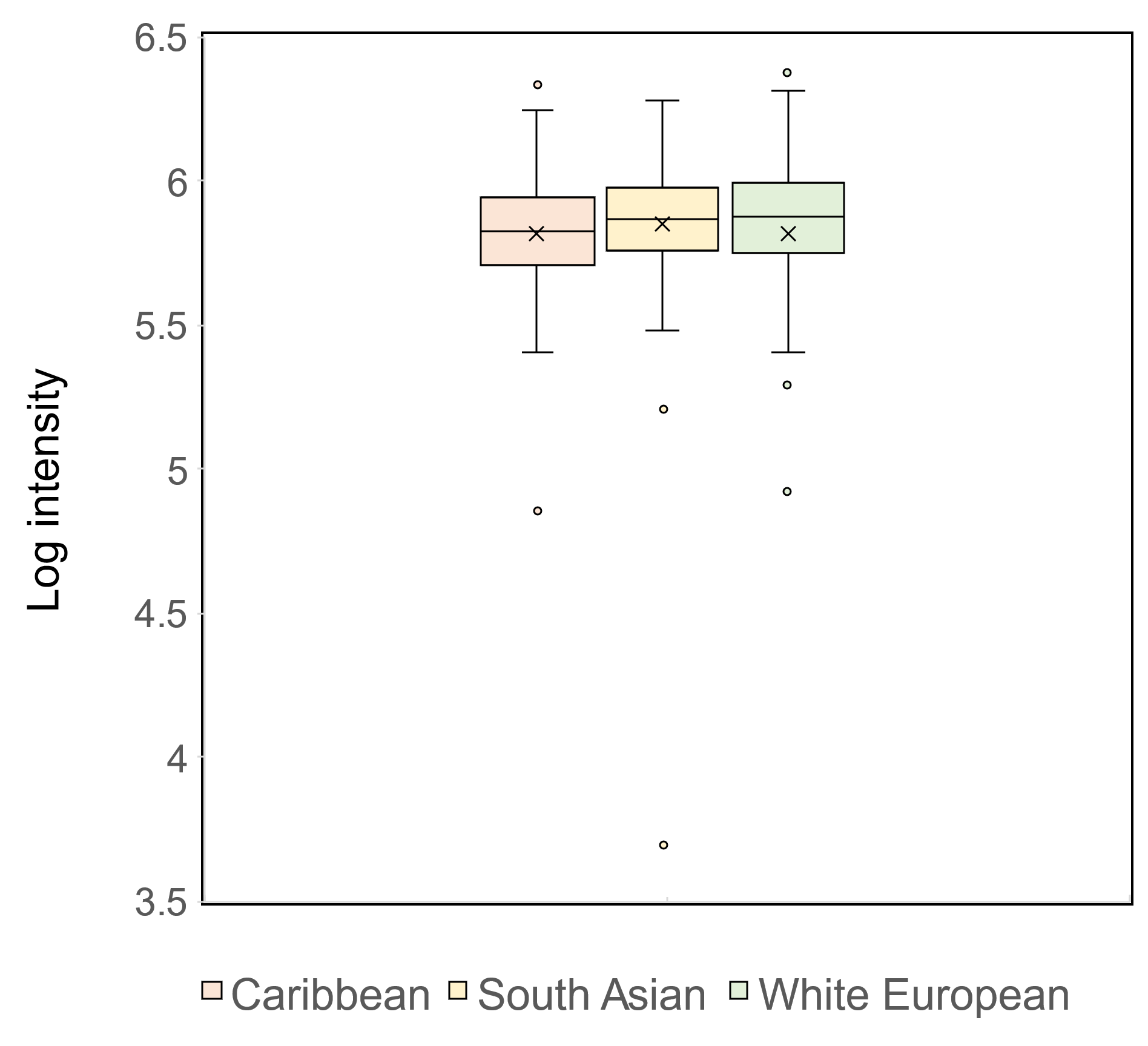


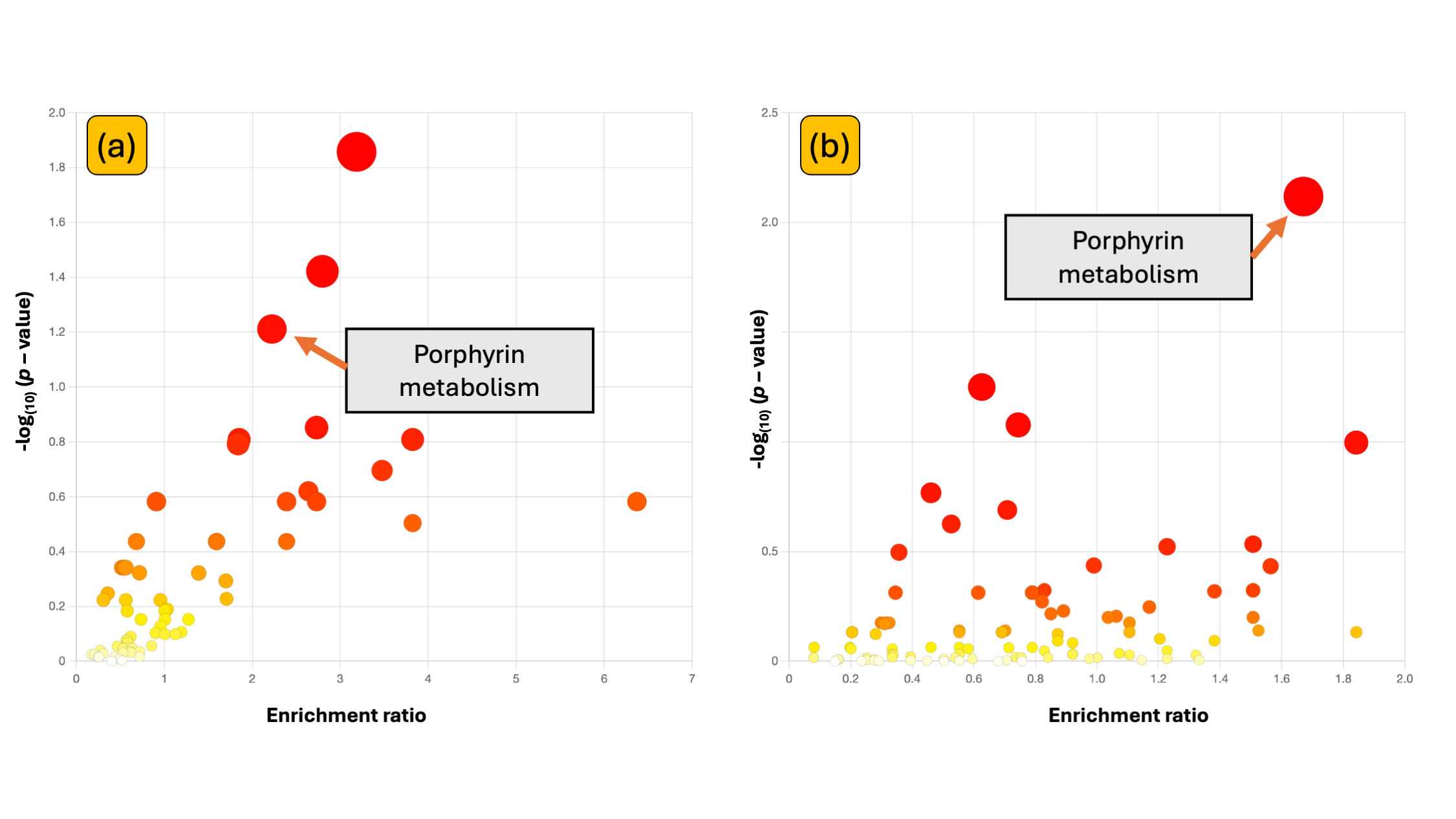


**Figure S10** Pathway enrichment analysis results from MetaboAnalyst 6.0. This functional analysis used significant m/z features, and their corresponding t-test scores, and p-values specific for age models built with (a) only drinkers and (b) only non-drinkers from the study population. This rules out the influence of alcohol intake in age specific pathway enrichment of Porphyrin metabolism reported in this study.

**Figure S9** Distribution plots for citric acids for the three ethnic groups investigated. X-axis indicates all ethnic groups i.e., Caribbean, South Asian and White European for which the abundance of citric acid was compared. Y-axis shows the log intensities of citric acid. No significant difference was observed in citric acid levels across ethnicities. This indicates that citric acid which has been previously reported to be associated with age in both males and females is in fact a potential metabolic indicator of age-progression and independent of an individual’s ethnic background. This should be further validated across other ethnicities in the future.

**Table S6** List of pathways enriched by age (A) or ethnicity (E) specific m/z features alongside total and significant compound hits with respective KEGG compound IDs.

| **Pathway name** | **Total hits on pathway** | **Significant no. of hits** | **KEGG compound IDs of**  **significant hits**  **on the pathway** |
| --- | --- | --- | --- |
| TCA cycle (A) | 10 | 4 | C00026;C05379;C00311;C00158;  C01352; C00417 |
| Porphyrin metabolism (A) | 16 | 8 | C15672;C00430;C05769;C05791;C01079;  C00500;C05770;C00486 |
| Biosynthesis of unsaturated fatty acids (E) | 17 | 8 | C01832;C00712;CE2246;C06429;C06428;C02679;C03242;C01595 |
| N-Glycan biosynthesis (E) | 5 | 5 | C04500;C01246;C00159;C04537;C00031 |
